# Blackfoot Words: a database of Blackfoot lexical forms

**DOI:** 10.1007/s10579-022-09631-2

**Published:** 2023-03-31

**Authors:** Natalie Weber, Tyler Brown, Joshua Celli, McKenzie Denham, Hailey Dykstra, Rodrigo Hernandez-Merlin, Evan Hochstein, Pinyu Hwang, Nico Kidd, Diana Kulmizev, Hannah Morrison, Matty Norris, Lena Venkatraman

**Affiliations:** Yale University, New Haven, USA

**Keywords:** Blackfoot, Algonquian, Language change, Language variation, Lexical database

## Abstract

This paper describes the structure and creation of Blackfoot Words, a new relational database of lexical forms (inflected words, stems, and morphemes) in Blackfoot (Algonquian; ISO 639-3: bla). To date, we have digitized 63,493 individual lexical forms from 30 sources, representing all four major dialects, and spanning the years 1743–2017. Version 1.1 of the database includes lexical forms from nine of these sources. This project has two aims. The first is to digitize and provide access to the lexical data in these sources, many of which are difficult to access and discover. The second is to organize the data so that connections can be made between instances of the “same” lexical form across all sources, despite variation across sources in the dialect recorded, orthographic conventions, and the depth of morpheme analysis. The database structure was developed in response to these aims. The database comprises five tables: Sources, Words, Stems, Morphemes, and Lemmas. The Sources table contains bibliographic information and commentary on the sources. The Words table contains inflected words in the source orthography. Each word is broken down into stems and morphemes which are entered into the Stems and Morphemes tables in the source orthography. The Lemmas table contains abstract versions of each stem or morpheme in a standardized orthography. Instances of the same stem or morpheme are linked to a common lemma. We expect that the database will support projects by the language community and other researchers.

## Introduction

This paper describes the structure and creation of Blackfoot Words, a new relational database of Blackfoot lexical forms (words, stems, and morphemes).^1^ Blackfoot (ISO 639-3: bla; Algonquian) is spoken by four indigenous Nations in Canada and the United States (Frantz, 2017; Mithun, 1999). Most fluent speakers are older and the language is rarely learned in the home by children (Genee & Junker, 2018). Now is the time to create language documentation which can help transmit Blackfoot language and culture to future generations. Our goal is to create a portable digital resource (Bird & Simons, 2003) which can support community-based language maintenance programs as well as research projects. The database structure emerged in response to the challenge of digitizing and organizing the data, which includes variation at multiple levels.

The paper is organized as follows. We discuss our aims and scope in Sect. 2, as well as how these differ from existing Blackfoot databases. In Sect. 3 we give some relevant background on the phonology and grammar of Blackfoot. In Sect. 4 we discuss the main sources of variation in our data, and in Sect. 5 we point out some of the challenges of working with this type of data. In Sect. 6 we describe the database structure we developed to address these challenges. We describe the methods we used to create the database in Sect. 7, with an emphasis on how we ensured consistency across the database. Finally, in Sect. 8 we point out some research projects that this database could support in the future, and in Sect. 9 we conclude.

## Aims and scope

There are two main aims of our database. Blackfoot is relatively well-documented, with language resources dating back to the mid-1700s. In aggregate, these contain a large amount of synchronic and diachronic lexical data, which could potentially be used in language projects. One barrier is that many of these sources are difficult to discover and access without an institutional library subscription. Given that, our *first aim* is to provide access to the lexical data in these sources for the research and speaker communities by digitizing the inflected forms contained within. Another barrier is the huge amount of variation across sources, which obscures the relationships between many lexical forms. Therefore, our *second aim* was to digitize these sources in a consistent format and to organize the data so that instances of the “same” lexical form could be grouped together across all of the sources. The resulting database structure emerged organically in response to these aims.

Version 1.1 of the database includes lexical forms from nine language documentation texts, including grammars, dictionaries, and wordlists. We digitized inflected word tokens in these textual materials, along with associated data such as translation, category, the phrase the word was found in, the maximal stem, the maximal stem lemma, and more. To date, we have digitized 63,493 individual lexical forms (tokens) from a total of 30 sources, representing all four major dialects, and spanning the years 1743–2017, which we plan to include in later versions. We digitized by token rather than type in order to preserve the data of the original sources as faithfully as possible, in accordance with our first aim. We also provide a morphemic analysis of each word token into morpheme tokens and multimorphemic stem tokens, and abstract lemmas of those morphemes and stems. Each abstract lemma is linked to by the individual tokens it derives from, allowing generalization across different orthographies and sources, in accordance with our second aim. We strived to create a flexible database structure which could be adapted to include other types of source materials in the future, such as transcripts of audio or video recordings, or interlinear glossed texts.

This type of diachronic corpus of forms is unique for Blackfoot. Two other lexical databases exist for Blackfoot, but these differ from Blackfoot Words in terms of scope and function. The first is the Blackfoot Digital Dictionary,^2^ which is part of the Blackfoot Language Resources project (Genee & Junker, 2018; Weilandt, 2018). The Blackfoot Digital Dictionary began as a way to adapt the most recent dictionary (Frantz & Russell, 2017) to a more user-friendly format, and it is maintained in collaboration and consultation with Blackfoot team members. Therefore, it necessarily prioritizes words as they are pronounced and used today, rather than including information about how these words have been written across many time periods and source materials. It also includes information which we do not, such as audio and video clips in definitions. The second synchronic lexical database is the Online Linguistic Database (OLD) for Blackfoot (Dunham, 2013, 2014).^3^ The OLD is a software for linguistic fieldwork that facilitates collaborative language documentation. The OLD allows users to build corpora of texts and provides tools for morpheme analysis, among other features. The sources included in the OLD include lemmas from the second edition of the dictionary (Frantz & Russell, 1995), as well as documentation from linguistic elicitation. One key way that Blackfoot Words differs from the OLD is that we document some of the hierarchical structure in individual tokens (e.g. stems and morphemes). There are ways that these three projects could build off of each other in the future. For example, it might be possible to feed some of the example words and phrases from Blackfoot Words into the Blackfoot Digital Dictionary. We could potentially use the morphological parsers in the OLD on the data from Blackfoot Words.

## Language background

This section briefly describes the phonology (Sect. 3.1) and grammar (Sect. 3.2) of Blackfoot. A grounding in *phonology* is necessary to understand the many orthographic notations included in the database, which vary in how well they capture phonemic contrasts and phonetic variation (discussed in Sect. 4.4). Regarding *grammar*, Blackfoot is a polysynthetic language and the morphosyntax is accordingly complicated, with a variety of different categories that combine in complex ways. Our aim in this section is not to give a complete grammatical overview of Blackfoot but to highlight a few properties which were a challenge for the database and which we discuss later, such as stem recursion and the internal complexity of verb stems. We also introduce some of the traditional Algonquian linguistic terminology that we use later in the paper, some of which are adopted in the database as category terms (discussed in Sect. 6).

### Phonology

The phonemic consonant inventory is given in Table 1 and the phonemic vowel inventory is given in Table 2 in IPA and in an orthographic representation using the most recent standardized orthography, designed by Donald Frantz and enumerated in Frantz (1978, 2017) and used in Frantz and Russell (2017). This orthography is an alphabet whose symbols map transparently to the phonemic inventory of the language. In brief, doubled letters represent long sounds, <y> is used for /j/, <'> for /ʔ/, <h> for /x/, <ai> for /ɛː/, and <ao> for /ɔː/. Note that the orthography also marks high f0 (one of the phonetic properties associated with stress) with an acute accent. On long vowels, <́V́́V> denotes a level high tone and <́VV> denotes a falling tone (Frantz, 2017, pp. 3–4).

**Table 1:** Blackfoot consonant inventory

	Labial	Coronal	Dorsal	Glottal
Stops	p pː <p> <pp>	t tː <t> <tt>	k kː <k> <kk>	ʔ <’>
Assibilants		${{\text{ts}}^{\vphantom{\lambda}}}^{\!\!\!\!\!\!\frown}\,\,{{\text{ts}}^{\vphantom{\lambda}}}^{\!\!\!\!\!\!\!\!\frown}$ː <ts> <tss>	${{\text{ks}}^{\vphantom{\lambda}}}^{\!\!\!\!\!\!\!\frown}$ <ks>	
Pre-assibilants		ˢt ˢtː <st> <stt>		
Fricatives		s sː <s> <ss>	x <h>	
Nasals	m mː <m> <mm>	n nː <n> <nn>		
Glides	w <w>	j <y>

**Table 2:** Blackfoot vowel inventory

	Front	Central	Back
High	i iː <i> <ii>		o oː <o> <oo>
Mid	ɛː <ai>		ɔː <ao>
Low		a aː <a><aa>	

It is uncertain whether some of the assibilants and pre-assibilants in Table 1 are actually phonemic. Plain coronal plosives regularly assibilate to $[{{\text{ts}}^{\vphantom{\lambda}}}^{\!\!\!\!\!\!\frown}]$ or [] before high front vowels and glides (Frantz, 2017; Weber, 2020). We assume this is a process of neutralization because the assibilants and plain plosives can both occur before other vowel qualities, potentially creating contrast (Weber, 2020). See Frantz (2017) and Weber (2020) for a more abstract analysis which assumes that $[{{\text{ts}}^{\vphantom{\lambda}}}^{\!\!\!\!\!\!\frown}]\,$ and [] are regular allophones of the plain coronal plosives in all contexts. The pre-assibilants [ˢt] and [ˢtː] only occur after front vowels, but are included here because they are minimally contrastive with [t] and [tː] in the same position (Weber, 2020). The sounds [k] and [$\,{{\text{ks}}^{\vphantom{\lambda}}}^{\!\!\!\!\!\!\!\frown}$] contrast before underlying vowels, including high front vowels and glides (Armoskaite, 2006; Weber, 2020), but neutralize to $[{{\text{ks}}^{\vphantom{\lambda}}}^{\!\!\!\!\!\!\!\frown}]$ before epenthetic [i] (Weber 2020, 2021, 2022). Long [] only occurs when a geminate /kː/ assibilates to [] before [i] in derived environments, and is not included in Table 1.

Table 2 includes five long vowels, /iː/, /ɛː/, /aː/, /ɔː/, and /oː/, and three short vowels, /i/, /a/, and /o/. Traditional descriptions of Blackfoot (e.g. Frantz, 2017; Kinsella, 1972; Taylor, 1969) argue that the long centralized mid vowels [ɛː] and [ɔː] are not contrastive because they only arise across morpheme boundaries: long [ɛː] arises from an underlying /a+i/ sequence, and long [ɔː] arises from an underlying /a+o/ sequence. However, these vowels occasionally occur morpheme-internally where they occur in the same environment as other long vowels (Weber, 2020; Weber & Miyashita, *forthcoming*). Note that the high back vowel varies in quality from a mid vowel like [o] to a high vowel like [u] (Weber, 2020).

All consonants except for [ʔ], [x], and the glides [w] and [j] have short and long counterparts, which are not always consistently distinguished in all sources. For all consonants except $[{{\text{ks}}^{\vphantom{\lambda}}}^{\!\!\!\!\!\!\!\frown}]$ this length distinction is contrastive. Example (1) gives a minimal pair which demonstrates that the length of a medial [t] is contrastive. Note that vowels are predictably short and centralized before geminate consonants, as in (1b). These represent the speech of Tootsinam (Beatrice Bullshields; BB), who does not pronounce the final orthographic <wa> sequence on verbs (Bliss & Glougie, 2010).

**Table:** 

(1)	a.	[ʔɛ́ːpo**t**aː]	*áípo****t****aawa*	‘he gets beat up’	(BB)
	b.	[ʔɛ́ːpʊ**t**ːaː]	*áípo****tt****aawa*	‘he is flying’	(BB)

Vowel length is contrastive for the vowel qualities [i], [a], and [o], as illustrated by the minimal pair in (2). Like consonant length, vowel length is not always consistently distinguished in sources.

**Table:** 

(2)	a.	[ʔâːk**o**kaː]	*aak****o****kaawa*	‘he will rope’	(BB)
	b.	[ʔâːk**oː**kaː]	*aak****oo****kaawa*	‘she will hold a Sundance’	(BB)

Several consonants have an unusually restricted or expansive distribution. The dorsal fricative /x/ only occurs before an obstruent (Elfner, 2006; Reis Silva, 2008; Weber, 2020). The glottal stop /ʔ/ typically occurs before a consonant of any kind (except for /x/), although occasionally between vowels, as in the vocative /naʔá/ ‘mother!’ (Peterson, 2004). The sibilants /s/ and /sː/ have an unusually wide distribution and can occur before, after, or between other consonants. No other consonants have this distribution (Goad & Shimada, 2014). Because of these distributions, the only true consonant clusters are /xC/, /ʔC/, and /sC/, and consonants or consonant clusters may additionally be followed by /j/. Not all sources consistently mark /x/ and /ʔ/ in these clusters.

There are several predictable realizations (allophones and neutralizations), which some orthographies mark and others do not. For consonants, there are the assibilants described above. Additionally, the velar fricative /x/ has allophones [ç] after front vowels and [xʷ] after round vowels (see Miyashita, 2018; Weber, 2020). For vowels, the long mid vowels before glottal stops are frequently diphthongized. Although the description of this allophony takes up considerable space in reference materials (Frantz, 1978, 2017, pp. 2–3; Kinsella, 1972; Lowery, 1979; Taylor, 1969; Uhlenbeck, 1938), there is considerable variation in pronunciation depending on the dialect, phonological context, and speaker (Weber & Miyashita, *forthcoming*). Long vowels shorten before /x/. Short vowels devoice before /x/; the result is often a highly fricated vowel (Miyashita, 2018). Short, centralized vowels [ɪ ʊ ɛ ɔ ɐ] occur in some types of closed syllables (Elfner, 2006; Weber, 2020). Note that the centralized low vowel [ɐ] is also transcribed as [ə] (e.g. Frantz, 1978) or [ʌ] (e.g. Denzer-King, 2012; Kaneko, 1999; Weber, 2020).

Some words end in a voiceless vowel or aspirated plosive (Bliss & Gick, 2009, 2017; Gick et al., 2012; Windsor, 2017a, 2017b; Prins, 2019). Voiceless word-final vowels are almost soundless and low in amplitude, contrasting sharply with the higher-amplitude of fricated vowels before /x/. Speakers will notice if you repeat a word back incorrectly and instruct you to look at their faces while they repeat the word. There is speaker variation in how these are phonologically and phonetically realized (Bliss & Glougie, 2010), and this variation is apparent in our sources as well.

Finally, each word in Blackfoot has at least one prominent syllable, and sometimes more than one (called “pitch accent” in Frantz, 2017). We follow Weber (2020) in analyzing prominence as the acoustic manifestation of primary stress. Stress is signaled primarily with a higher f0, as well as greater intensity and duration (Van Der Mark, 2003). The location of the pitch peak on nouns is not predictable (Stacy, 2004; Weber, 2020; Weber & Shaw, 2022), but the location of the pitch peak on some types of verbs is predictable, and displays rhythmic metrical properties, such as obligatoriness, culminativity and edge demarcation (Weber, 2016, 2020). Stress can fall on any syllable of the word, except for syllables with voiceless nuclei.

### Grammar

Blackfoot is a polysynthetic language, which is strongly head-marking (Bliss, 2013; Louie, 2015) with morphologically complex “clausal” words (Weber, 2020, 2021, 2022). This can be illustrated with the basic morphological template for the verbal complex, in (3) below (Bliss, 2013; Frantz, 2017; Taylor, 1969; Weber, 2020). We focus the discussion here on verbs. However, the nominal complex is also head marking, with either mono- or multimorphemic stems (see Bliss, 2013; Kaneko, 1999; Kim et al., 2017; Ritter & Rosen, 2014; Wiltschko & Ritter, 2015). The stem (in square brackets below) can be preceded by a person prefix and any number of optional prefixes known as “preverbs,” which have a wide variety of grammatical functions. The stem is followed by obligatory inflectional suffixes (marking person and clause type), which differ for intransitive versus transitive verbs, and optional DP enclitics.

**Table:** 

(3)	*Morphological Template of the Verbal Complex*
	person–(preverb*)–[initial–(medial)–final]–suffixes (=clitics).

Example (4) illustrates this template with a verb which contains a complex stem and several preverbs. As shown here, preverbs cover a wide range of meanings and grammatical functions, including tense, aspect, and modality (TAM) markers, negation, quantifiers, relative roots (which introduce oblique DPs), and modifiers of various kinds.^4^ Preverbs do not occur in a fixed order; instead, the linear order corresponds to semantic scope meaning (Bliss, 2010, 2013: 13*ff*). Third person arguments typically have a null person prefix, as shown in this example.

**Table:** 

(4)	áakotoowahsoohpommaawa	
	aak–oto–√owahsi–[√ohpomm–aa]–Ø–wa	
	fut–go.to–√grub–[√buy–ai]–ind–3	
	‘she will go grocery shopping’	(Frantz & Russell, 2017, p. 212)

The Blackfoot stem is itself morphologically complex and contains at least two elements, named ‘initial’ and ‘final’ for their position in the stem (Armoskaite, 2011; Bliss, 2013; Bloomfield, 1946; Déchaine & Weber, 2018; Goddard, 1990). The initial is minimally a √root, and the finals derive stem classes based on transitivity and animacy. A bipartite intransitive stem is shown in (5a), where the inanimate intransitive (II) final *-ii* agrees with the inanimate subject. The initial and final may be separated by a medial, which is typically a √root with a nominal or classifying meaning (Biedny et al, 2021; Dunham, 2009; Frantz, 2017; Goddard, 1990). For example, (5b) includes a stem with the same initial and final as in (5a), plus the medial *-kom-* ‘liquid’.

**Table:** 

(5)	a.	áaksiksístoyiwa	b.	áaksiksístokomiwa	
		aak–[√ksisto–yi]–Ø–wa		aak–[√ksisto–**kom**–i]–Ø–wa	
		fut–[√warm–ii]–ind–3		fut–[√warm–√**liquid**–ii]–ind–3	
		‘it will be warm’		‘it will be warm water’	(Frantz & Russell, 2017, p. 64)

The same underlying morpheme may occur in different positions within this template. For example, *omahk-* ‘big’ is an initial in (6a) but a preverb in (6b). These examples also show that roots and stems form derivational paradigms (Bauer, 1983, 1997), which we return to in Sect. 5.3.

**Table:** 

(6)	a.	ómahksimma	b.	ómahksiníkkssapiwa	
		[**√omahk**–i]–mm–a		**√omahk**–√inikk–[√ss–api]–Ø–wa	
		[**√big**–ai]–ind–3		**√big**–√sulking–[√thus–look.ai]–ind–3	
		‘he is older’		‘she gave a [big—NW] sulking glance’	(Frantz & Russell, 2017, pp. 190, 73)

Finally, stems can be recursive when the verbal complex contains a compound or a so-called “secondary derivation” (Goddard, 1990), where a full stem is followed by another final.^5^ In example (7), the reflexive final *-ohsi* derives an animate intransitive (AI) stem from a bipartite transitive animate (TA) stem *ino* ‘see’. As indicated by the square brackets, this means that a TA stem is contained inside of an AI stem, creating a recursive stem structure.

**Table:** 

(7)	nitáínoohsspinnaan	
	nit–a–[[√in–o]–ohsi]–hp–innaan	
	1–ipfv–[[√see–ta]–refl.ai]–ind–1pl	
	‘We (excl.) see ourselves.’	(Frantz, 2017, p. 117; reglossed)

In sum, a verbal complex contains several different kinds of morphological constituents: the inflected word, one or more recursive stems, and morphemes of various kinds. There are different category types at each of these levels: word category is determined by syntactic and morphological distribution; stem category is determined by derivational morphology (e.g. by the finals); and morpheme category is traditionally determined by position within the template (e.g. as a preverb, initial, final, etc.). The database includes a standardized set of categories at each level, as we discuss in Sect. 6.2.

## Data

This section describes the sources and lexical data which form the core of the database. We focus on several points of variation within the data which affected our database design. The database includes documentation of all four main *dialects*, which are mutually intelligible, but which differ in some ways in terms of lexical items and pronunciation. Blackfoot *speakers* also exhibit many different ways of speaking which cross-cut the main dialectal divisions. The *sources* are a variety of types, including wordlists, dictionaries, grammars, and research articles. The *orthography* varies across and within sources due to a number of factors.

### Dialects

Blackfoot is the westernmost language of the Algonquian family, spoken in southern Alberta, Canada and northern Montana, USA (Mithun, 1999, pp. 336–337; Frantz, 2017).^6^ It is the language of four distinct indigenous Nations that share a common language, culture, and heritage. Three Nations are in Canada: Siksiká (Blackfoot), Káínai (Blood), Aapátohsipikani (Peigan, or Northern Peigan). The fourth Nation, Aamsskáápipikani or Piikúnni (Blackfeet, or Southern Piegan),^7^ is located in the USA.^8^ The two Piikáni (Piegan) Nations separated in the 1800s (Hungry Wolf & Hungry Wolf, 1989, p. 3) and have had different political histories since then due to existing in two different countries. Together, the four Nations form the Blackfoot Confederacy^9^ (Siksikaiitapi), which is an alliance of solidarity rather than a centralized governing body (Dempsey, 2019; Grinnell, 1892, p. 153; Juneau, 2007, 13*ff*).

**Fig. 1:**
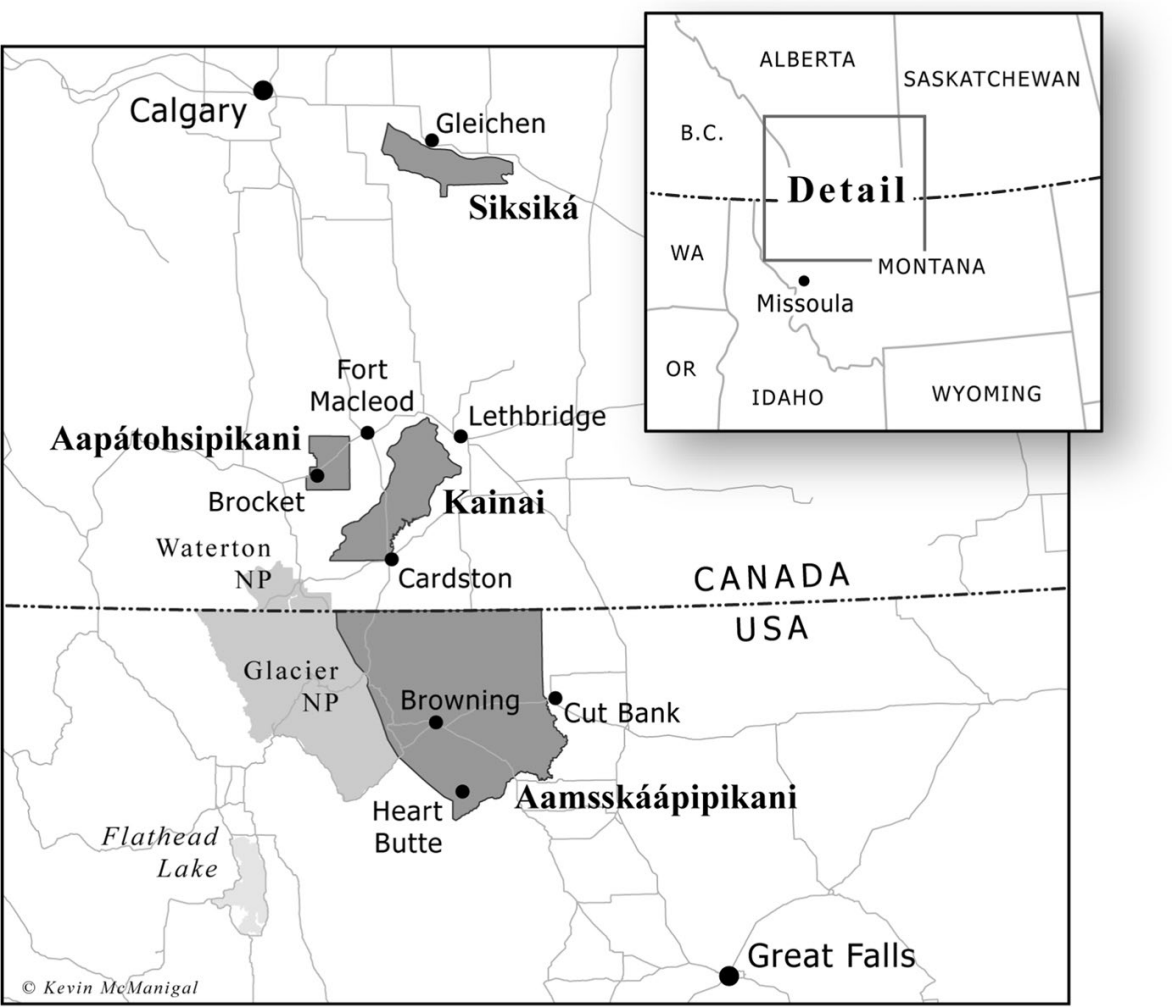
Locations of Blackfoot reserves. Map by Kevin McManigal

Each of the four Blackfoot Nations today is associated with a separate reserve (in Canada) or reservation (in the USA), shown in gray in Fig. 1. The pre-contact Blackfoot territory was much larger, ranging over areas of modern-day Alberta, Saskatchewan, Idaho, and Montana (Dwyer & Stout, 2012; Genee & Junker, 2018; Hungry Wolf & Hungry Wolf, 1989). The Siksiká (Blackfoot) reserve is slightly southeast of Calgary near Gleichen. The Aapátohsipikani (Peigan, or Northern Peigan) reserve is located at Brocket, southwest of Fort Macleod, Alberta. The Káínai (Blood) reserve is southeast of the Aapátohsipikani, near Cardston and Stand Off, Alberta. The Aamsskáápipikani (Blackfeet, or Southern Piegan) reservation is located in northwest Montana in Glacier County. Many Blackfoot people also live off-reservation (Genee & Junker, 2018).

The language as a whole is typically known in English as ‘Blackfoot’ in Canada, or ‘Blackfeet’ in the United States.^10^ The language is also known as Siksiká (which means ‘Blackfoot’ or ‘Blackfeet’ in the language itself), or similar translations in other languages (e.g. Pied-Noir in French). Each of the four Nations is associated with a particular dialect, which are mutually intelligible but contain minor differences in lexical items and pronunciation (Bliss & Ritter, 2009; Peter, 2014, p. 9, 14*ff*; Frantz, 2017, 2*ff*; Frantz & Russell, 2017, p. *xiii*). When speaking English, the four dialects associated with the four Nations may also be referred to by the name of the Nation. The name Siksiká is therefore associated with the language as a whole as well as the name of the northernmost dialect. (This dialect is sometimes called “proper Blackfoot,” perhaps because it is the same as the language name.) Within the language itself, a common endonym is Niitsí’powahsin (‘Real/True/Original Language’). Speakers may also refer to the individual dialects associated with each Nation (i.e. Siksikáí’powahsin, Káínai’powahsin, Piikáni’powahsin, Aamsskáápipikani’powahsin, and Aapátohsipikani’powahsin).

Table 3 summarizes the Blackfoot and English names of the language and the four dialects (numbered), along with their locations, glottocodes, and ISO codes. The metonymic usage of Siksiká to refer to the language as a whole is reflected in the glottocodes (from Glottolog; Hammarström et al., 2021) associated with the language and dialects, shown in Table 3. Glottocodes are assigned to each unique *languoid*, defined in Cysouw and Good (2013) as any language-like object, such as a family, language, or dialect. The glottocode for the language as a whole is siks1238, while the Siksiká dialect does not have its own separate glottocode. The Káínai and Piikáni dialects each have a separate glottocode, but the two Piikáni subdialects are not distinguished. To avoid this confusion, we refer to each dialect in the database by a combination of their Blackfoot and English names. We have also listed in Table 3 the Blackfoot ISO code (from Ethnologue; Eberhard et al., 2021). These are assigned to each unique language, which Ethnologue defines based on a number of factors, including linguistic similarity, intelligibility, a common literature, speaker views, and more. All four dialects of Blackfoot are mutually intelligible, so it is unsurprising that there is a single ISO code for the language.

**Table 3:** Relation between language and dialect names, glottocodes, and ISO codes

Dialect (Blackfoot)	Dialect (English)	Country	Glottocode	ISO Code
Siksiká (language)	Blackfoot	Canada/USA	siks1238	bla
1. Siksiká (dialect)	Blackfoot	Canada	(none)	n/a
2. Káínai	Blood	Canada	bloo1239	n/a
Piikáni	Piegan	Canada/USA	pieg1239	n/a
3. Aapátohsipikani (or Piikúnni)	Northern Peigan	Canada	(none)	n/a
4. Aamsskáápipikani	Blackfeet, or Southern Piegan	USA	(none)	n/a

The descriptive literature focuses on salient differences between the four dialects, but little is known about the extent of variation within a single dialect. All four dialects are represented in the database, which means that the database could be used to study differences between and within dialects, as we discuss in Sect. 8.1.

### Speakers

According to national census tracking, Blackfoot is spoken by 2750 people in southern Alberta, Canada (Statistics Canada, 2017) and another 1450 in the United States (U.S. Census Bureau, 2015). As Genee and Junker (2018) point out, it is difficult to determine the degree of fluency among self-reporters, as well as the amount of under- or overreporting that is represented in these numbers. They estimate that there are likely fewer than 5000 reasonably fluent speakers, representing perhaps 15% of the ethnic Blackfoot population, most of whom are over the age of 50 (Statistics Canada, 2011, 2012, 2017; U.S. Census Bureau, 2015; Genee & Junker, 2018, pp. 277–278). The language is classified as “shifting” in Canada and “moribund” in the United States (according to Ethnologue; Eberhard et al., 2021), with fewer than 100 speakers in Montana. Despite this, Blackfoot remains an important component of cultural transmission. It is used in traditional ceremonies, and there are Blackfoot language programs in Canada and the United States. However, the census numbers suggest that it is only a matter of decades before the remaining fluent elders have passed on, and it will become significantly more difficult to transmit the language to future generations.

Many different speakers are represented in the database, which means that the database could be used to study differences between speakers of the same or different dialects. The database could also potentially support projects in language maintenance and pedagogy, which we discuss further in Sect. 8.5.

### Sources

Blackfoot is relatively well-documented, and there are several primary references (grammars and dictionaries) of Blackfoot. Of these, the most recent are the *Blackfoot Grammar* (Frantz, 1997, 2009, 2017), and the *Blackfoot Dictionary of Stems, Roots and Affixes* (Frantz & Russell, 1989, 1995, 2017). Both of these draw on earlier resources from the twentieth century, such as earlier grammars (Taylor, 1969; Uhlenbeck, 1938), dictionaries by Uhlenbeck and Van Gulik (1930, 1934), and written and oral texts (De Josselin de Jong, 1914; Uhlenbeck, 1911, 1912). For a summary of these major works of documentation, as well as a summary of more recent work on Blackfoot linguistics, stories, and more, see Genee and Junker (2018).

There is also a long chronology of older documentation dating back to the mid-eighteenth century, much of which is documented in Taylor (1969). Many of these resources were compiled by missionary linguists, anthropologists, or explorers working for fur trading companies. These older resources are not easily accessible because most are out of print and/or copyright, and exist primarily as bound copies. Furthermore, these older resources are not always discoverable by researchers without prior knowledge of their existence. For example, they are not listed in aggregator sites like Glottolog (Hammarström et al., 2021) or WALS (Dryer & Haspelmath, 2013), both of which are often used in the early stages of research to discover language resources. The earliest source in the Glottolog entry for Blackfoot is Howse (1849), and the earliest source in the WALS entry is Uhlenbeck (1938). One of the goals of this project is to make these resources more discoverable and accessible (Bird & Simons, 2003) by digitizing the lexical forms in them.

The database draws from a variety of published sources on Blackfoot, spanning just under 300 years. All of these sources were published, which means that the project did not need to digitize handwritten field notes or manuscripts nor decipher handwriting, which has been an issue for other lexical databases of similar time depth (cf. Baldwin et al., 2016; Bowern, 2016). The earliest source is a list of numbers collected by James Isham around 1743 (published 1949; cited in Taylor, 1992; see Brasser, 1979, p. 35), although the most extensive early source is the vocabulary of Umfreville (1790).^11^ The most recently published source is Frantz (2017). Table 4 includes a list of the sources whose lexical forms have been digitized at the time of writing, organized by date of publication. Not all of these sources are included yet in the database; we include an overview of the sources and their completion in Sect. 7.5. The table includes the number of individual records found in each resource to provide an approximation of their contribution to the database. We continue to add to this list. For example, we plan to include the most recent published version of the *Blackfoot dictionary of stems, roots, and affixes* (Frantz & Russell, 2017) in future versions of the database.

**Table 4:** Current sources with digitized words

Reference	Resource type	Number of records
Isham (1949/1743)	Wordlist	10
Umfreville (1790)	Wordlist	45
Franklin (1823)	Wordlist	21
Hale (1846)	Wordlist	~141
Catlin (1842)	Wordlist	~137
Latham (1846)	Wordlist	77
Gallatin (1848)	Wordlist	107
Hale (1848)	Wordlist	~170
Howse (1849)	Wordlist	~364
Schoolcraft (1854)	Wordlist	~236
Hayden (1863)	Grammar and wordlist	~1458
Morgan (1871)	List of kinship terms	~415
Lanning (1882)	Grammar and dictionary	~3941
Hale (1886)	Wordlist	90
Lacombe (1886)	Reader	~1330
Tims (1889)	Grammar and dictionary	~9270
Wilson (1888)	Wordlist	~255
Maclean (1896)	Grammar	~1339
Curtis (1911)	Wordlist	445
Geers (1917)	Dictionary	~2473
Schultz (1926)	List of topographical feature names	~203
Uhlenbeck and van Gulik (1940)	Dictionary	~15,890
Uhlenbeck (1938)	Grammar	~14,328
Voegelin (1941)	Article on Blackfoot's place in the Algonquian family	~177
Schultz (1962)	Book of stories about Blackfoot life	~593
Taylor (1967)	Article on morphophonology	152
Taylor (1969)	Grammar	3605
Frantz (1971)	Linguistic grammar	~1476
Holterman (1996)	Dictionary	~2265
Frantz (2017)	Learners' grammar	~2480
Total=30 sources		~63,493

The authors of these sources include—to the extent that they can be categorized—fur traders and travelers (e.g. James Isham, Edward Umfreville, Joseph Howse); priests of various denominations (e.g. Albert Lacombe, John Tims); people interested in ethnology and philology (e.g. Horatio Hale, Robert Latham, James Willard Schultz) and professional anthropologists (e.g. Lewis H. Morgan, John Maclean); people who engaged in linguistic scholarship during their work and travels in North America (e.g. Edward S. Curtis, Ferdinand V. Hayden); linguists (e.g. Albert Gallatin, Gerardus Geers, Christianus Uhlenbeck, Robert van Gulik, Charles Voegelin, Allan Taylor, Jack Holterman, Donald G. Frantz); and amateur enthusiasts (e.g. Cash M. Lanning, a jeweler, gunsmith, and silversmith in Fort Benton, MT, who bought a small printing press and self-published a grammar and dictionary of Blackfoot).

Regarding dialects and speakers, virtually all sources in the database with 1000 or more records document the Aamsskáápipikani (Southern Piegan) dialect; with the exceptions of Tims (1889) and Lacombe (1886), both of which document Siksiká (Blackfoot), and Maclean (1896), which documents the Káínai (Blood) and, to a lesser extent, the Aapátohsipikani (Northern Peigan) dialects (Brownstone, 2008). It is not yet clear which dialect or dialects were documented in Hayden (1863), although the author appears to have worked in the United States primarily. Little, if any, is known about the particular Blackfoot speakers who contributed to these sources, and only a few authors (e.g. Frantz, 1971; Frantz & Russell, 2017; Lanning, 1882; Schultz, 1926; Taylor, 1969) name their Blackfoot consultants. Occasionally an author publishes words obtained from another researcher. For example, both Gallatin (1848) and Latham (1846) publish parts of a wordlist given to them by Kenneth McKenzie, a trader for the American Fur Company.

A large timespan is represented in the database, which means that the database could be used to study changes across time.

### Orthography

Because Blackfoot was traditionally not written down, early missionaries and researchers employed a wide range of constructed orthographies (Genee, 2020, pp. 4–6). All of the sources included in the initial publication of the database use symbols from the Latin alphabet, albeit often with one or more diacritics, such as <ḣ> or <ế>.^12^ However, sources vary widely in (a) whether they reflect the phonemic (contrastive) sounds of the language, (b) phonetic accuracy, and (c) internal consistency. We discuss some examples of these types of variation below.

Sources differ in their phonemic accuracy. The orthography in Frantz (1978, 2017) and Frantz and Russell (2017), which we employ in this paper, captures all phonemic contrasts and also explicitly writes some predictable sounds, like the assibilants $[{{\text{ts}}^{\vphantom{\lambda}}}^{\!\!\!\!\!\!\frown}]$ <ts> and $[{{\text{ks}}^{\vphantom{\lambda}}}^{\!\!\!\!\!\!\!\frown}]$ <ks>. The orthography in Taylor (1969) also captures all the contrastive sounds of the language, albeit with a slightly different set of symbols than Frantz (e.g. <x> instead of Frantz's <h>). Sources prior to Taylor (1969) frequently missed contrastive sounds, especially consonant and vowel length, and pre-consonantal consonants. This is illustrated by the set of words in (8). As shown by the orthographic transcriptions from Frantz and Russell (2017), Blackfoot contrasts short (8a) and long (8b) consonants, and the long consonants are distinct from clusters of a glottal stop followed by a consonant (8c,d) or a dorsal fricative followed by a consonant (8e). However, even quite extensive earlier works like Uhlenbeck (1938) and Tims (1889) do not reliably transcribe these contrasts.

**Table:** 

(8)		Frantz and Russell (2017)	Uhlenbeck (1938)	Tims (1889)	Gloss
	a.	mo**k**sísa	mo**k**sís	mo**k**sĭs’	‘awl’
	b.	mó**kk**oyisi	mo**k**úyis	mo**k**u’yĭs	‘fur’
	c.	mo**’k**sísi	*(not given)*	mo**k**sĭs’si	‘armpit’
	d.	mo**’t**sísi	mo**t**sís	mo-**t**sĭs’	‘hand’
	e.	mo**hk**ínsstsisi	mo**χk**ínistsis	mo**k**ĭns’tsĭs	‘elbow’

Sources differ in their phonetic accuracy. The sources which are more phonemic often do not write down predictable segments or positional variants. For example, <h> in Frantz's (1978, 2017) orthography represents the phoneme /x/ regardless of how it is actually realized. This phoneme is realized as [ç] after front vowels, [xʷ] after rounded vowels, and [x] elsewhere (Miyashita, 2018; Weber, 2020). On the other hand, Uhlenbeck (1938) transcribes two separate dorsal fricatives, “*x* being the palatalized variant of *χ* after *i*, or diphthongs with *i* as second component” (Uhlenbeck, 1938, p. 2). Similarly, older sources often distinguish far more vowel qualities. In most cases, these distinctions are unlikely to be phonemic and simply reflect what the transcriber perceived. However, given that the Blackfoot vowel system has undergone several mergers and splits (Berman, 2006; Oxford, 2015), these differences could reflect true distinctions that existed at earlier stages of the language.

In some cases the orthography reflects the underlying forms, creating morphological transparency at the expense of phonetic accuracy. For example, when a morpheme that ends in /a/ precedes a morpheme that begins in /i/, the underlying sequence /a+i/ can be pronounced as [ɛː] or [ej] or [aj], depending on factors like dialect and phonological context (Frantz, 1978, 2017, pp. 2–3, 183). However, Frantz (1978, 2017) and Uhlenbeck (1938) spell /a+i/ invariably as <ai>, regardless of whether this is pronounced as a monophthong or a diphthong. Other sources use a spelling which is more accurate to the pronunciation, which reveals dialectal and free variation. Example (9) below includes a word with an underlying sequence /aj/ (9a), a word with an underlying /a+i/ sequence across a morpheme boundary (9b), and a possibly underlying mid vowel in (9c) which Frantz and Russell (2017) spells variably with <aai> or <aii>. Maclean (1896), who recorded the Káínai (Blood) and Aapátohsipikani (Northern Peigan) dialects, uses <ai> for all three words, suggesting that all have the same pronunciation [aj]. However, Lacombe (1886), who recorded the Siksiká (Blackfoot) dialect, transcribes the diphthong in (9a) with <ay> but the other two words in (9b,c) with <e>, possibly representing a monophthongal mid vowel. Thus, these older orthographies can reveal dialectal differences because they are more phonetically accurate than the phonemic or broad phonetic orthographies from Frantz (1978) and Taylor (1969).

**Table:** 

(9)			Frantz and Russell (2017)	Maclean (1896)	Lacombe (1886)	Gloss
	a.	/aj/	**ááy**o’kaawa	**Ai**okao	**ay**okaw	‘he sleeps’
	b.	/a+i/	kan**áí**tapiwa	kûn**ai**tûpĭ	kan**e**tapix	‘all people’
	c.	/eː/	n**áái**pisstsiwa	N**ai**pĭstcĭ	N**e**pistsi	‘blanket, cloth’

Vowel length transcription is a less obvious example where orthography often reflects underlying forms rather than the actual pronunciation. Frantz's (1978, 2017) and Taylor's (1969) orthographies reflect vowel length contrasts, except in several phonological positions where vowel length is variable, or difficult to distinguish. Both authors write vowels as long at the end of the word or before glottal stops if there are morpheme alternations showing that the vowel is long in other contexts or if the vowel is composed of two underlying short vowels (Taylor, 1969, pp. 35–36; Frantz, 2017, p. 6). The vowel is written as short if there are morpheme alternations showing the vowel is short, but in cases where there is no evidence either way, such as for a morpheme-internal vowel before a glottal stop, the vowel tends to be written as if it is long (Taylor, 1969, pp. 35–36). These transcriptions are misleading, because they do not reflect the actual pronunciation of the word but *appear* to do so based on the fact that the orthographies otherwise accurately transcribe vowel length.

Not all sources are internally consistent. Longer sources often included a key which explains the relation between orthographic symbols and sounds. These sources are usually internally consistent in how they spell. In general, older and shorter sources contain less consistent notations, which either result from a one-to-many or a many-to-one relationship between sounds and symbols. For example, Frantz and Russell (2017) employs a unique orthographic sequence for short vowels (10a), long vowels (10b), and a voiced vowel followed by a dorsal fricative (10c). Catlin (1842) records each of these three sounds or sequences of sounds with multiple orthographic notations (for example, in (10a) a short vowel can be represented by either <ah> or <a>). He also uses a single orthographic notation to represent multiple sounds; for example, <ah> is employed for short vowels (in *Ahtsaiks* ‘Leggings’), long vowels (in *Ahkeoquoin* ‘Girl’), and a vowel followed by a dorsal fricative (in *Sah komape* ‘Boy’).

**Table:** 

(10)		Frantz and Russell (2017)		Catlin (1842)	
	a.	n**a**tsííks	‘my pants’ [with *n-* ‘my’]	**Ah**tsaiks	‘Leggings’
		**a**sóka’simi	‘clothing, usu. a jacket or overcoat’	**A**ssokas	‘Shirt’
	b.	**aa**kííkoana	‘girl’	**Ah**keoquoin	‘Girl’
		o’tok**áá**ni	‘her hair’	Otok**a**n	‘Head’
	c.	s**aah**kómaapi	‘boy’	S**ah** komape	‘Boy’
		pisst**ááh**k**aa**ni	‘tobacco’	Pist**a**c**a**n	‘Tobacco’
		**ááh**siiwa	‘it is/was good’	**Ahgh**see	‘Good’

Several sources use a particular way of transcribing vowels that is based on English spelling rules. These sources separate clusters of letters by a hyphen or space, and a single vowel symbol is used to represent two distinct sounds, depending on whether they are followed by a consonant in the same cluster or not. Hayden (1863) uses this system to distinguish centralized and peripheral vowels. For example, if the symbol <i> is followed by a consonant, it represents a centralized vowel [ɪ], as in (11a). If it is not followed by a consonant, it represents a peripheral vowel [i], which may be short, (11b), or long, (11c). The vowel in (11a) is followed by a geminate consonant, as shown by the transcription in Frantz and Russell (2017). Centralization is not represented in Frantz's (1978, 2017) orthography because vowels are predictably centralized in certain positions (such as before the geminate consonant in (11a)) and are otherwise peripheral.^13^ Note that Hayden's orthography is truly tracking vowel quality and not syllabification or the presence of geminate consonants. For instance, he also follows the first vowel in ‘dog’ (11b) with a consonant <m>, suggesting that he heard a centralized vowel here, but that <m> is not a geminate consonant, as shown by the spelling in Frantz and Russell (2017).

**Table:** 

(11)			Frantz and Russell (2017)		Hayden (1863)	
	a.	[ɪ]	nitáán**i**kka	‘he told me’	ni-ta’-n**i**k	‘he told me’
	b.	[i]	im**i**tááwa	‘dog’	im’-**i**-ta’-o	‘dog’
	c.	[iː]	p**íí**taawa	‘eagle’	p**i**-ta’	‘eagle’

Lanning (1882) uses a similar system, although he additionally mapped English vowel qualities to these letters. As he put it, a letter without a following consonant has “the sound of its English name” (Lanning, 1882, p. 5). For example, the symbol <i> in (12a) represents [aj] at the end of a cluster, but [ɪ] when followed by a consonant. The symbol <e> in (12b) represents short or long [i] at the end of a cluster, but [ɛ] when followed by a consonant. The symbol <o> in (12c) represents short or long [o] at the end of a cluster, but [a] when followed by a consonant. And [ɐ], which is the actual centralized counterpart of [a] in Blackfoot, is represented instead by <u>, (12d). Lanning (1882) also uses many other strategies to transcribe these sounds which do not follow the pattern in (12); however most map transparently from English spelling rules (e.g. <neese> sounds like English “niece”, or <oak> sounds like English “oak”).

**Table:** 

(12)			Frantz and Russell (2017)		Lanning (1882)	
	a.	[aj]	**áy**immiwa	‘he’s laughing’	**I** im e oo	‘he is laughing’
		[ɪ]	nitáán**i**kka	‘he told me’	nit A n**i**k	‘he told me’
	b.	[i]	im**i**tááwa	‘dog’	e m**e** tA	‘a dog’
		[iː]	p**íí**taawa	‘eagle’	p**e** tA	‘an eagle’
		[ɛ]	**áí**mmóniisiwa	‘otter’	**e**m o neese	‘otter’
	c.	[o]	pon**o**káwa	‘elk’	po n**o** kA	‘elk’
		[oː]	m**oo**kítsisa	‘toe/finger’	m**o** ke tis	‘finger’
		[a]	apin**á**kosi	‘tomorrow’	ap pe n**o**k wis	‘tomorrow’
	d	[ɐ]	iss**á**mmisa	‘look at him!’	s**u**m mis	‘look at him!’

Many orthographic notations are used in the database, representing varying degrees of phonetic and phonological accuracy. This means that the database could potentially be used to study phonological variation and change. However, this type of study is complicated by inconsistent orthographies in some sources, as well as a non-trivial mapping between orthographies across sources.

## Challenges and decisions

Because we were working with a diverse set of sources, the data (described in Sect. 4) includes a lot of variation. In this section we describe some of the challenges for such varied data in terms of *tokenization*, *phonemicization*, and *lemmatization*. The solution we implemented in the final database structure includes tokens of words, stems, and morphemes (given in the original source orthography) linked to stem and morpheme lemmas (our own abstractions and analyses, given in a standardized orthography).

### Tokenization

There are several challenges in “chunking” the data into useful subunits, a process known as tokenization (Grefenstette, 1999). Sources vary in what size of unit they include: phrases, words, stems, morphemes. We opted to use the inflected word as our largest token size, so we broke phrases down into individual words and assigned them their own translation. This distinguishes us from databases where phrases are only translated as a whole, such as Brixey and Artstein (2021). Not all sources demarcate words with a unique delimiter, like a space or hyphen. Sometimes a word delimiter is also used to separate substrings of words. In other cases, two inflected words are not separated by a word delimiter. Because of this variation in how word delimiters were used, we tokenized by hand rather than using an automated method. This method requires a knowledge of Blackfoot inflectional morphology in order to determine where word boundaries fall.

An example of a word delimiter being overused is shown below, where a space occurs after the first syllable of a word, (13), or after some word-internal prefixes, (14). We always included affixes in our word tokens, even when they were separated by a word delimiter, as in (14).

**Table:** 

(13)	a.	Sah komape	‘Boy’	Catlin (1842, p. 264)
	b.	O makatôse	‘God’	Lacombe (1886, p. 71)

**Table:** 

(14)	a.	o sinâkisin	‘his book’	Lacombe (1886, p. 63)
	b.	Ki kata enowâwex?	‘Do you see them?’	Lacombe (1886, p. 49)

An example of a word delimiter being underused is shown below. Umfreville (1790) separates syllables by a hyphen. However, the entry in (15a) includes two words. Examples (15b,c) show both of these words individually, as cited from the recent dictionary, Frantz and Russell (2017); (15c) is even inflected for a possessor. This also applies to certain other phrasal combinations, such as common demonstrative-noun collocations. Example (16) shows that the demonstrative *ánnohk* ‘now’ (to use the orthography from Frantz, 1978, 2017) is sometimes written separately and sometimes together with ‘day’ in a phrase meaning ‘today’. We always split entries like (15a) and (16b) with multiple inflected words into multiple records in the database.

**Table:** 

(15)	a.	Meek-shim-no-coce	‘A Pot’	Umfreville (1790, p. 202)
	b.	mí’ksskimma	‘metal (object)’	Frantz and Russell (2017, p. 151)
	c.	nóóhko’sa	‘my dish’	Frantz and Russell (2017, pp. 139)

**Table:** 

(16)	a.	Anuqk tcĭstcĭkwe	‘To-day.’	Maclean (1896, p. 160)
	b.	anookchusiquoix	‘to-day’	Latham (1846, p. 37)

There is also variation across and within each source regarding whether phonological clitics are written as separate orthographic words or part of the following word. This is true for the [wh] marker *tsa*= (Barrie, 2014), the conjunction *ki*=, and bare demonstrative stems like *am*= ‘this’ or *om*= ‘that’. Example (17) shows that some sources write *tsa*= ‘wh’ as a separate word, (17a), while others write it together with the following word, (17b). For these types of phonological clitics, we tokenized the word in the same manner as the original source author; e.g. (17a) was tokenized as two words and (17b) was tokenized as a single word. As we discuss below, all words were further tokenized into stems and morphemes, so the internal complexity of the word tokens in (17b) was captured in other ways in the database.

**Table:** 

(17)	a.	tsa kitau'-an-i?	‘What are you saying’	Tims (1889, p. 11)
	b.	Tsâkitawâni?	‘What do you say?’	Lacombe (1886, p. 84)

A final issue is that stems and morphemes occur in different derived contexts across different sources. For example, the morpheme *omahk-* ‘big’ occurs in two different stems in (6). In some cases, a stem may *only* occur in derived contexts within a source, even though it occurs in underived contexts in other sources. This is the case for ‘garment’, which occurs in underived contexts with two stem variants: one with an initial [a], (18a), and one without (18b). However, there are also sources which *only* contain this stem as part of a derived stem, (19) and (20), where the stem which means ‘garment’ is underlined. Without further analysis, the connection between the examples in (18)–(20) would be lost.

**Table:** 

(18)	a.	Assokas	‘Shirt’	Catlin (1842, p. 263)
		a-sú-kas-sĭm	‘shirt’	Curtis (1911, p. 170)
		asókaaʔsimʔi	‘coat, shirt, garment’	Taylor (1969, p. 47)
		asóka’simi	‘clothing (jacket or overcoat)’	Frantz and Russell (2017, p. 18)
	b.	soo kos	‘a shirt’	Lanning (1882, p. 68)
		su’-kōs	‘coat’	Hayden (1863, p. 267)
		sokásimi	‘shirt’	Geers (1917, p. 48)
		sókàsimi	‘shirt’	Uhlenbeck (1938, p. 60)
		sokasĭm	‘an outer garment or a coat’	Maclean (1896, p. 129)

**Table:** 

(19)	*Sources where the stem for ‘garment’ only occurs with prenoun* isttohk-*‘thin’*		
	xistokisokasim	‘shirt’	Holterman (1996, p. 40)
	istox’isokŏsǐm	‘shirt’	Tims (1889, p. 165)
	E-stoke-so-char-sim	‘A Shirt’	Umfreville (1790, p. 202)

**Table:** 

(20)	*Sources where the stem for ‘garment’ only occurs with prenoun* staaht- *‘under’*		
	Starsi-sokâs	‘Shirt’	Lacombe (1886, p. 77)

To meet these challenges, we decided to tokenize at several different linguistically relevant levels: the word, the stem, and the morpheme. This circumvents the problem of word or morpheme demarcation across sources. Regardless of whether some authors analyze phonological clitics as part of another word or as a separate word, and regardless of whether a stem occurs in derived or underived contexts, all components will be tokenized down to constituent stems and morphemes, all in the original source orthography. This lets us build some of the hierarchical structure of words into the database. By comparing these tokens *across* sources, we are in a much better position than previous researchers to determine lemmas for each token.

### Phonemicization

As we discussed in Sect. 4.4, there are many different orthographic systems used across the database. In some cases, two written forms look so different that it is not obvious they are transcriptions of the same lexical form. Ideally, the database would provide a way to standardize and phonemicize the data, so that instances of the same forms look alike. Many other databases which face similar challenges in orthographic variation provide a phonemicized version of each word token in a standardized orthography (cf. Baldwin et al., 2016; Bowern, 2016). We opted against this method of phonemicization for several reasons.

First, very few of our sources use phonemicized orthographies, which means that standardizing the source orthographies would rarely be a simple conversion from one phonemicized orthography to another. We would have to make non-trivial decisions about how to resolve ambiguous notations, especially for sources which use inconsistent or non-phonemic transcriptions. In some cases a source that uses a phonemic orthography may include an obviously related form which could serve as the basis for phonemicization. For example, (21c) could be used as the phonemicized form for (21a,b) because Frantz and Russell (2017) uses a standardized orthography which captures all phonemic contrasts, as we discussed in Sect. 4.4. In other cases, like (22), there are no obvious related forms in contemporary resources, so it is unclear how it should be phonemicized.

**Table:** 

(21)	a.	Ohkitchis	‘Fingers’	Catlin (1842, p. 264)
	b.	okutshish	‘nails’	Gallatin (1848, p. *cxiii*)
	c.	ookítsisi	‘her toe, finger’	Frantz and Russell (2017, pp. 45, 121)

**Table:** 

(22)	a.	oh kittakes	‘hand’	Gallatin (1848, p. *cxiv*)
	b.	Okittakis	‘hand’	Latham (1846, p. 35)
	c.	*(no obvious related form in *Frantz & Russell, 2017*)*

Second, many authors failed to transcribe some sounds, which means we would have to decide whether to correct these omissions, and if so, how. An illustrative example involves dialectal variants of the word for ‘earth’, (23). The database includes some examples of ‘earth’ with an initial $[{{\text{ks}}^{\vphantom{\lambda}}}^{\!\!\!\!\!\!\!\frown}]$, (23a), and others with an initial $[{{\text{ts}}^{\vphantom{\lambda}}}^{\!\!\!\!\!\!\frown}]$, (23b). This reflects known speaker variation between a so-called t-dialect and a k-dialect (Peter, 2014: 15; Uhlenbeck, 1938, p. 6), where speakers of the t-dialect may use [ts] in place of some $[{{\text{ks}}^{\vphantom{\lambda}}}^{\!\!\!\!\!\!\!\frown}]$. The database also includes transcriptions like (23c), which fail to capture the initial assibilant. If we provided a standardized orthographic transcription for each word token, we would need to decide whether (23c) “truly” began with $[{{\text{ks}}^{\vphantom{\lambda}}}^{\!\!\!\!\!\!\!\frown}]$ or $[{{\text{ts}}^{\vphantom{\lambda}}}^{\!\!\!\!\!\!\frown}]$, when both are valid possibilities.

**Table:** 

(23)	a.	**ks**ááhkomma	‘earth’	Frantz and Russell (2017, p. 140)
		**ks**ahcoom	‘earth’	Latham (1846, p. 36)
		**ks**áχkum	‘earth’	Geers (1917, p. 15)
		**kr**ahkum	‘earth, world, land’	Holterman (1996, p. 8)
		**ks**ŏk'kum	‘Earth’	Tims (1889, p. 129)
	b.	**tch**ok kome	‘earth, dirt’	Lanning (1882, p. 67)
		**te**haqkoum	‘earth’	Hale (1886, p. 702)
		**Ts**arkoum	‘Earth-globe’	Lacombe (1886, p. 71)
		**Ts**aqkom	‘Earth, land’	Maclean (1896, p. 133)
	c.	**s**uḣ'-um	‘earth’	Hayden (1963, p. 268)

Third, standardizing the orthography also runs the risk of misinterpreting true synchronic or diachronic differences as transcription mistakes, thereby sanitizing the data of interesting variation. This risk is especially pronounced because our data comes from so many different time periods and dialects. Example (24a) includes three different tokens of ‘beaver’ from sources from the 1800s in the database, compared to a modernized spelling representing synchronic pronunciation in (24b). This word is a reflex of the Proto-Algonquian (PA) stem in (24c), which includes the initial *ki·šk- ‘chop’ (Bloomfield, 1946, pp. 86, 114, 120) and transitive inanimate (TI) final *-ant ‘by mouth’ (Bloomfield, 1946, p. 113; Proulx, 1989, p. 60), followed by a common Blackfoot animate intransitive (AI) final *-aki* and inflectional *-wa* ‘3’.

**Table:** 

(24)	a.	**K**ekstakee	‘beaver’	Catlin (1842, p. 263)
		**k**akestake	‘beaver’	Latham (1846, p. 36)
		**k**ikkistakew	‘the beaver’	Lacombe (1886, p. 27)
	b.	**ks**ísskstakiwa	‘beaver’	Frantz and Russell (2017, p. 141)
	c.	PA *ki·šk-ant-	‘chop by mouth’

Note that the initial sound for each token in (24a) is [k] (bolded), which is closer to the PA form in (24c) than the current pronunciation in (24b). If these correspondences are regular across all forms, this kind of data would show that a regular sound change of PA *k > *ks* before *i or *iˑ (Berman, 2006, p. 265) occurred in this word quite recently in the late 1800s or early 1900s. (All three sources contain words with an orthographic sequence that represents $[{{\text{ks}}^{\vphantom{\lambda}}}^{\!\!\!\!\!\!\!\frown}]$, so it is not the case that they simply failed to transcribe this sound.) The tokens in (24a) also differ in whether they record an overt vowel between [k] and [st] (underlined). The current pronunciation in (24b) does not have a vowel in this position, while the PA form in (24c) does. It is possible that this variation represents historical variation, where some dialects had already undergone vowel deletion in this position while others had not. It is also possible that all dialects had undergone vowel deletion, and some transcribers simply misheard the [kst] sequence. Finally, the forms in (24a) also use a variety of vowel symbols which may or may not represent true differences in quality or length. If we provided a standardized orthographic transcription for each word token, we would need to decide whether to standardize to the modern pronunciation or not on each of these points.

Ultimately, we opted to leave all words in their original orthographies and leave analysis to future researchers. Instead, we decided to link all tokens of the “same” stem or morpheme to an abstract, standardized lemma. That way, the database itself imposes no analysis on the original transcriptions, but does provide lemmas as a way for researchers to view all tokens of the same form at once. The precise mechanism for “linking” stems and morphemes is deeply tied to the problems of tokenization, discussed in Sect. 5.1 above, and lemmatization, discussed in Sect. 5.3 below.

### Lemmatization

Broadly construed, a lemma is a common base form among many tokens. As discussed in Knowles and Mohd Don (2004), “lemma” is not well-defined, and has been used with a variety of definitions, including: an ad hoc group of words, a dictionary headword or citation form, a set of inflectional variants, a label for a paradigm or set of paradigms, the name of a set of lexical items, or a set of words including spelling variants. In Blackfoot Words, we primarily use lemmas as the name of a set of all instances of that lemma across all sources, regardless of orthographic, dialectal, or diachronic variation.

We lemmatize at the stem and morpheme levels, but not at the word level. One common type of lemma groups words into inflectional classes by lemmatizing at the stem level. For example, Francis and Kučera (1982, p. 1) define the lemma as “a set of lexical forms having the same stem and belonging to the same major word class, differing only in inflection and/or spelling”. However, Blackfoot stems create problems for lemmatization because they are multimorphemic, and consist minimally of a root (an initial) and a final (a bound derivational suffix) (Déchaine & Weber, 2015, 2018). (The internal structure of stems was discussed in Sect. 3.2 Grammar.) The stem-internal morphemes form derivational paradigms (Bauer, 1983, 1997), and as a result there are far more Blackfoot stems than their English equivalents. To take the stems in (5) as an example, ‘be warm’ and ‘be warm water’ would be listed as separate stems in Blackfoot, although they both contain the same initial *ksisto-* ‘warm’ and the same II final *-i*. (See Kazeminejad et al., 2017 for a concise explanation of the same problem for Arapaho and Arkhangelskiy & Lander, 2015 for Adyghe.) Because the main lexical meaning in both stems is contributed by the root, the root would also be a natural lemma, as it is in Semitic languages (Knowles & Mohd Don, 2004). Since we already tokenize each stem and morpheme and since we wanted to create phonemicized forms for each token, we decided to create lemmas at both levels: lemmas at the stem level essentially create a set of all words in an inflectional paradigm; lemmas at the morpheme level create a set of all stems in a derivational paradigm. In other words, the purpose of the lemma is to provide an abstract record which links all instances (tokens) of that lemma together.

Each lemma contains standardized, abstract, dictionary-type information. The translation is a short, abstract English gloss, which is useful especially when some sources give incorrect glosses or use languages other than English for their translations. The form of the lemma is given in Frantz's (1978, 2017) orthography, which captures all phonemic contrasts. The lemma form abstracts away from allomorphy. Morphemes may have different morphophonological shapes depending on their position in the word (e.g. as a preverb versus an initial) or local phonological context (e.g. after a consonant versus a vowel). As an example, all instances of the stem meaning ‘garment’ from (18)–(20) would be grouped under one lemma, with examples shown in (25) below. Here, we arbitrarily chose a lemma with an initial [a]. This groups together all instances of this stem, including variants with and without that initial [a], as well as a few instances which only occur in derived contexts and which begin with an initial [i] in that position. Searching for the lemma would bring up all of these instances and more, allowing researchers to study morphophonological generalizations in Blackfoot (see Sect. 7.3).

**Table:** 

(25)	**Lemma:** *aso’kasim* ‘garment (dress, shirt, coat)’					
	a.	Assokas	‘Shirt’		Catlin (1842)	(= 18a)
	b.	soo kos	‘shirt’		Lanning (1882)	(= 18b)
	c.	isokasim	‘shirt’	(as part of *xistokisokasim*)	Holterman (1996)	(= 19)
	d.	i-sokâs	‘shirt’	(as part of *Starsi-sokâs*)	Lacombe (1886)	(= 20)

The lemma form also abstracts away from dialectal and orthographic variation. As an example, all instances of the stem meaning ‘Earth’ would be grouped under a single lemma, shown in (26). Here, we chose a lemma with an initial $[{{\text{ks}}^{\vphantom{\lambda}}}^{\!\!\!\!\!\!\!\frown}]$, following the most recent dictionary (Frantz & Russell, 2017). However, the lemma groups together the dialectal variants with an initial $[{{\text{ks}}^{\vphantom{\lambda}}}^{\!\!\!\!\!\!\!\frown}]$ or $[{{\text{ts}}^{\vphantom{\lambda}}}^{\!\!\!\!\!\!\frown}]$, as well as instances which failed to capture the initial sound of this word. This way, we are not forced into phonemicizing each individual instance, but a search for this lemma would clearly indicate that (26c) is an instance of the lemma for ‘Earth’.

**Table:** 

(26)	**Lemma:** *ksaahkomm* ‘Earth’					
	a.	ksááhkomm	‘earth’	(as part of *ksááhkomma*)	Frantz and Russell (2017, p. 140)	(= 23a)
	b.	tchok kome	‘earth, dirt’		Lanning (1882, p. 67)	(= 23b)
	c.	suḣ'-um	‘earth’		Hayden (1963, p. 268)	(= 23c)

To summarize, we tokenize each word form down to the stem and morpheme levels. Each lemma allows us to group all instances of a stem or morpheme into a set to form inflectional and derivational paradigms. We are able to phonemicize the lemmas at linguistically-relevant levels (e.g. stem and morpheme) without needing to make judgments about the best phonemicization of each individual instance of that lemma in the corpus, which we leave for future research.

## Database structure

This section describes the database software and structure. The database design emerged organically as the project progressed in response to the challenges we discuss in Sect. 4. The database is designed to capture the *hierarchical* structure of stems, as well as *abstract* relationships between stems and their constituent parts.

### Software

In order to create a portable digital resource (Bird & Simons, 2003), the database is fully open. All of the software used to create and display the database is open-access, and the full database and dataset is also open-access and downloadable.

The database was created using MySQL, an open-source relational database management system. To accommodate the variety of symbols, the database uses a unicode font encoding. The data is hosted on a dedicated serverless MySQL (5.7) database, which is based on Amazon AWS Aurora Serverless v1. The advantage of a serverless database is that it automatically pauses the database during periods of inactivity, starts up on demand, and automatically scales memory and CPU resources based on utilization. This database was created in Yale Spinup, a web portal providing on-demand creation and management of virtual servers, databases, and storage through Amazon AWS and Storage@Yale. Spinup resources are deployed in the cloud behind Yale University's enterprise firewall, and exposed to the wider internet via an additional web proxy.

The database can be viewed at https://www.blackfootwords.com/view/. Version 1.1 of the database is displayed using NocoDB, a free, open-source platform that turns any database into a smart spreadsheet. Users must register with a free account to view the database in NocoDB. The data may also be downloaded from a Zenodo repository at https://doi.org/10.5281/zenodo.5774980. This repository includes the entire MySQL schema and data, created via mysqldump.

All relational databases contain multiple tables of information with links between certain parts of the tables.^14^ Each table contains multiple entries, called records, which each have a unique identifier, called a key. Tables also contain fields, which are types of data, and each record may have a different value for each field. A visual representation would treat records as the rows of a table, fields as the columns of the table, and field values as the information in each table cell.

**Fig. 2:**
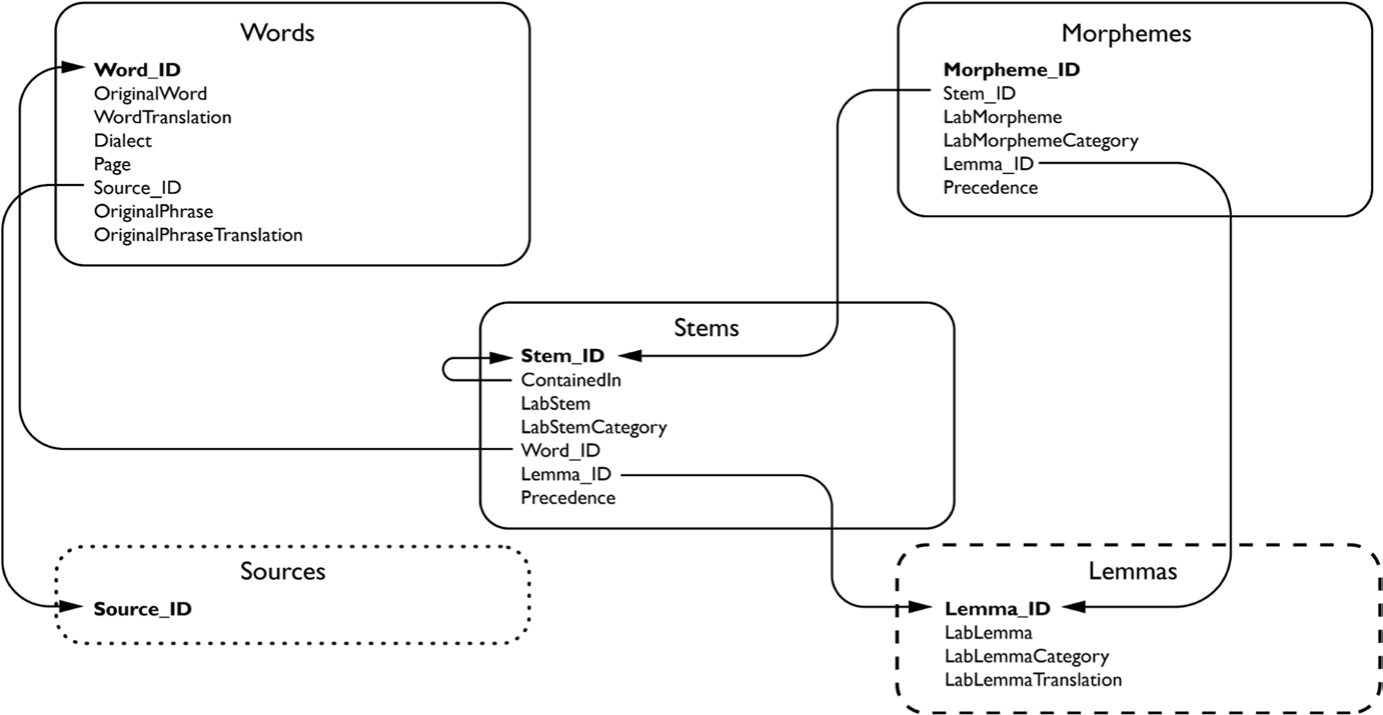
Links between tables. Only a subset of fields is shown in each table. The Tables outlined in a solid line (Words, Stems, and Morphemes) contain fields in the original source orthography (e.g. OriginalWord, LabStem, and LabMorpheme). The table outlined in dashes (Lemmas) contains fields in a standardized orthographic representation (e.g. LabLemma). The table outlined in dots (Sources) contains bibliographic information about each of the sources

The Blackfoot Words database consists of five main tables: Sources, Words, Stems, Morphemes, and Lemmas. Figure 2 illustrates the main links between tables. Note that the Words, Stems, and Morphemes tables (solid outlines in Fig. 2) include individual tokens, not types. If the same word, stem or morpheme appears more than once across sources, then it will have a separate entry for each appearance. Only the Lemmas table (dashed outline) is what might be considered a traditional dictionary. The reason is that each instance of a word often occurs with different metadata, e.g. a word repeated within a grammar might have a slightly different translation each time, and words which are part of examples within dictionary entries occur in slightly different contexts each time. We opted to create a faithful record of each instance of a lexical form, even when this resulted in many redundant entries. In this way, the Words table faithfully records primary source material without imposing any analysis on it.

The Words, Stems, and Morphemes tables preserve the hierarchical structure of each Blackfoot word token in the following way. Each inflected word token is entered into “OriginalWord” in the Words table using the source orthography. These words are then analyzed into constituent stems and morphemes, preserving the source orthography.^15^ These constituent parts are entered as records in the Stems table (in “LabStem”) or the Morphemes table (in “LabMorpheme”). Each stem and morpheme record contains a foreign key—meaning the key originates in another table—that relates it to the word or stem record it is contained in. This portion of the database was created via an iterative process (from inflected words, to stems, to morphemes), described in Sect. 7 below.

The Lemmas table contains abstract, standardized representations of the stems and morphemes. Each stem or morpheme record also contains a foreign key relating it to a lemma record. In this way, there is a many-to-one relationship between Stems and Lemmas and between Morphemes and Lemmas, and each morpheme or stem “is an instance of” a lemma. The Lemmas table is necessary because it allows a search to bring up related forms of the same stem or morpheme despite the number of distinct orthographic representations, which cannot be easily standardized (see Sects. 4.4 and 5.2). Instead, each lemma abstracts over multiple instances of the same stem or morpheme which have differences in form due to orthographic notations, phonological context, and synchronic or diachronic variation.

Throughout the database, we note which fields include information from the original source and which include our own analysis by using “prefixes” in the field names. Fields which begin with “Original” have values which come from the original source. Fields which begin with “Lab” have values that come from our analysis. The field “WordTranslation” has neither prefix, because some values are from the original source while others are from the lab, as described in Sect. 6.2.2 below. Each key is marked by a trailing “_ID” in the field name.

The following sections describe each table in more detail.

#### Sources

The Sources table includes bibliographic information for each source and other notes that help the reader to interpret that source. The Source_ID is an abbreviated identifier, typically the initials of the author's name followed by a four digit year. For sources with multiple authors, each author's initials are separated by an underscore. Type is the bibliographic type in BibLaTeX. The other fields are named after fields in BibLaTeX (Kime et al., 2020), such as “Author”, “Year”, “Title”, “Pages”, etc.

- Source_ID: An abbreviated source name, equivalent to a BibTeX key, that serves as a unique key for this source.
- Type: e.g. book, article, etc.; equivalent to a BibLaTeX type
- etc.

We also include comment fields about the dialect, orthography, and provenance of the source. The Dialect field notes the dialect recorded (when known). Many times, the person who wrote these lists was not very exact about who they were speaking with. For example, for those who documented Blackfoot as spoken in Canada, they did not always say which dialect each word is from (or whether there were any differences). In those cases, we use the country name rather than the dialect name. If the author included an orthographic key, then we copied this into the Orthography field. This information is necessary to interpret the ever-present variation in orthographic representations of Blackfoot between authors; for example, the vowel /ɛ/ (the vowel in the English words *bet* and *bed*) is transcribed as <ai> in Frantz and Russell (2017), <ɛ> in Taylor (1967), and <e> in Lanning (1882). The Provenance field includes notes about the original source of the data, broadly construed. For example, many sources reprint words from another researcher's fieldnotes, which we note here. Finally, there is an open comment field for any other notes from the lab.

- Dialect: Notes about the dialect recorded, if known.
- Orthography: Notes about the orthographic transcription.
- Provenance: Notes about the original source of the data (e.g. specific speakers or another researcher's journals).
- LabSourceComments: Any other notes by the lab.

#### Words

The sources in the database are of a variety of types (wordlists, dictionaries, grammars) with different amounts of notes, details, and formatting. For example, a grammar might include tables of inflectional suffixes, while dictionaries include a mix of stems (as entry headers) as well as inflected words and even phrases (as examples in the entries). There are thus a range of possible units of analysis, ranging from individual morphemes to entire phrases. We standardized across these sources by using lexical phonological forms as the unit of record in the Words table. By “lexical” we mean something the size of an inflected word or smaller, as described in Sect. 5.1. By “phonological form” we mean some kind of orthographic representation of pronunciation, rather than a representation of another aspect of language, such as a semantic concept. This also means that we did not include grammatical information or other comments that were not directly related to a lexical form.

The core type of lexical form in the database is the inflected phonological word. Each instance of a word is given a unique Word_ID. There is a set of fields to record other types of information associated with the word. Where possible, we have added dialectal information to the Sources table. However, we have also included a “Dialect” field in the Words table, because some sources include words from multiple dialects. We document the word in the source orthography (“OriginalWord”), as well as the word translation, category, and underlying representation, if given. If the original source contains a translation in a language other than English, we copy it verbatim using the source language. This is in accordance with our first aim to digitize and preserve the source material. Each word token is linked to a stem token, which in turn is linked to an abstract lemma. The lemma record contains an abstract translation in English. Most sources did not include a category (part of speech), and those that did used a wide variety of terms. Because of that, we also include a LabWordCategory, which assigns each word a category from a standardized, finite set. In practice, this set of categories is still in flux—it changes as the project progresses and as we encounter new word categories. We give a few typical examples below. An underlying representation (UR) represents an analysis by the original source author, and is a type of linguistic representation that abstracts away from some predictable aspects of pronunciation. URs are not typically found in dictionaries and grammars, but do occur in some linguistic grammars and articles.

- Word_ID: Unique key for each word entry.
- Source: Source_ID [foreign key linking to source record]
- Page: The page number where the word or phrase is found.
- Dialect: The source dialect of the word.
- Speaker: The speaker who contributed the lexical form.
- OriginalWord: A fully inflected word, given in the source orthography.
- WordTranslation: The translation of the word. If the word occurs as part of a phrase, then the translation was created by the lab. Otherwise, the translation is copied verbatim from the original source, no matter the original source's metalanguage.
- OriginalCategory: The word category or part of speech from the original source.
- OriginalUR: The underlying representation of the word from the original source.
- LabWordCategory: The word category or part of speech as determined by the lab. Examples: N (noun), V (verb), or D (demonstrative).

If a word occurs as part of a phrase, then we record the entire phrase and the phrase translation (into OriginalPhrase and OriginalPhraseTranslation, respectively). We filled in the WordTranslation ourselves, based on the phrasal context. That means that any word which has a non-empty OriginalPhrase field will have a translation which was created by the lab and not original to the source.

- OriginalPhrase: The phrase a fully inflected word occurs in.
- OriginalPhraseTranslation: The translation of the phrase a fully inflected word occurs in.
- OriginalPhraseUR: The underlying representation of the phrase from the original source.

There are also fields which record any notes on “partial words”, by which we mean anything smaller than a full inflected word, including sequences of affixes, stems, or combinations of stems and other morphemes.^16^ Partial words occur in many different fashions across the sources. For example, a dictionary entry header is always a morpheme or a stem; grammars may list strings of inflectional affixes in tables or columns; and authors of grammars and linguistic articles might include notes about parts of a word, such as a stem, while describing topics like Blackfoot grammar and historical changes in pronunciation. If a partial word was discussed with respect to a particular inflected word, we included it in the PartialWord fields of the inflected word's record. We did not prioritize consistency for the PartialWord fields in the same way we did for OriginalWord. There were many different types of PartialWords and associated information across the sources, and the values of the PartialWord fields are not in a standardized, consistent format.

- OriginalPartialWord: A partial word, given in the source orthography.
- OriginalPartialWordTranslation: The translation of the partial word from the original source.
- OriginalPartialWordCategory: The partial word category or part of speech from the original source
- OriginalPartialWordUR: The underlying representation of the partial word from the original source

Occasionally, a source discusses an underlying representation or a partial word without reference to an inflected word, or with reference to many inflected words (as for a dictionary entry with multiple inflected words in the entry examples). We digitized these as a separate record with an empty OriginalWord field, as long as they were associated with either a translation, category, or another lexical form (e.g. an underlying representation or a partial word). Partial words are therefore represented in two different ways in the database: (1) as part of a word record which has an OriginalWord, and (2) as part of a word record with an empty OriginalWord field. Both types of partial words are essentially a morphemic analysis of words by a particular author.

We include partial words in accordance with our first aim of digitizing and providing access to the lexical data in Blackfoot sources. At this time, we have not linked either type of partial word to a Lemma record. However, we can imagine research projects that could utilize this type of data, and the option to integrate partial words into the database structure remains open in the future.

Finally, there are several notes fields.

- CitedFrom: The original source of any words that are being cited from another source.
- OriginalComments: Comments from the original source that relate to one or more of the other fields.
- LabComments: Comments from lab members. These often note illegible words, probable typos, and any uncertainty regarding translations or other fields.

#### Stems

The Stems and Morphemes tables impose a morphemic analysis on each record from the Words table. The Stems and Morphemes records are where we, the lab, tokenize each inflected word by determining where morpheme breaks occur and what category each constituent part is. However, we leave these forms in their original orthographies.

Each stem is assigned a unique Stem_ID. The Stems table is the only table which allows a record to link to another record within the same table. This allows us to capture some of the hierarchical structure of the stem. The ContainedIn field contains a key which can link back to another record within the Stems table. The Precedence field contains a number which counts the place of the stem with respect to any sister stems and morphemes within the same constituent. (An example is given at the end of this section.) As explained in Sect. 6.2, the LabStem and LabStemCategory fields both have “Lab” prefixes because they represent an analysis by the lab. The LabStemCategory consists of a finite number of categories, which are more specific than the set of LabWordCategory values because they define stem classes in terms of animacy and transitivity. This set of categories is still in flux, but we give a few typical examples below. The Lemma_ID contains a foreign key linking to a record within the Lemma table. The Stem record does not contain a “translation” field; since all translations of stems are abstract, we opted to put the translation into the Lemma record.

- Stem_ID: Unique key for each stem entry.
- Word_ID [foreign key that links to the word that the stem is contained in]
- ContainedIn [foreign key that links to the stem the stem is contained in]: This field is NULL if the stem is the maximal stem, and otherwise it links to the stem that contains this stem.
- Precedence: This field is NULL if the stem is the maximal stem. For any stem contained in another stem, this field contains a number representing the linear order of the stem with respect to any other sister stems and morphemes.
- LabStem: The form of the stem in each word or stem token, given in the original orthography, as analyzed by the lab.
- LabStemCategory: The stem category or part of speech, as analyzed by the lab. Examples: NA (animate noun), NI (inanimate noun), VAI (animate intransitive verb), VTA (transitive animate verb), etc.
- Lemma_ID [foreign key that links to the abstract lemma record]: This allows us to link all of the different spellings of a stem or morpheme to a single unique lemma.
- LabStemComments: Any comments from lab members.

For the purposes of the database, each stem record is one of two types. The first is what we call a “maximal stem”, which is created by “stemming” an inflected word; that is, by removing the person prefix and all inflectional suffixes and DP enclitics. What remains is any number of preverbs plus a (possibly recursive) stem—in other words, lexical and derivational components. Our “maximal stem” is equivalent to a *stem* in computational linguistics (Bauer, 1983, pp. 21–22; Sproat, 1992, p. 249), because it is the base which can combine with inflectional affixes. The “maximal stem” does not correspond to a “stem” in Algonquianist terminology, but we have included it in the Stems table for three reasons. First, this helped us figure out the morphemic analysis of each stem, as described in Sect. 7.4. By moving all maximal stems into a single spreadsheet, it was easier to sort all words with a particular preverb together, and then to remove that preverb into the Morphemes table. Second, the maximal stem represents the first abstract level where it makes sense to create a Lemma. That way, if two sources have instances of the same complex stem which happen to be inflected for different persons or clause types, the stem Lemma will still group both instances together. Third, by defining Stems in this way, it means that every morpheme is contained inside a stem. (If we had not defined it this way, then some morphemes would be contained inside of words, and others inside of stems.) The second kind of stem in the Stems table corresponds to the “stem” in Algonquianist terminology (Bloomfield, 1946; Goddard, 1990). The Algonquian “stem” is essentially a minimal *stem* in computational linguistics, because it is the smallest base which can combine with inflectional morphology (Bauer, 1983, pp. 21–22; Sproat, 1992, p. 249).

As an example, here is a word from Tims (1889) in the source orthography, with a subset of the fields from the Words table shown.

**Table:** 

(27)	Word_ID:	word-JT1889-7010
	OriginalWord:	nitŭs’sŭmmosi
	WordTranslation:	‘I see myself’

After removing the only inflectional affix, namely nit- ‘1’, the structure is as follows. The maximal stem contains a morpheme, *ŭ-*, and another stem, (28a). This stem contains another (minimal) stem (the Algonquian “stem”), and another morpheme, *-osi*, (28b). The (minimal) intransitive stem is shown in (28c), and can be decomposed into two morphemes: the initial *s’s-* and the final *-ŭmm*. At each level of recursion, we give each constituent a precedence number with respect to the surrounding constituents.

**Table:** 

(28)	a.	Max stem:	[ŭ-	[s’sŭmmosi				]]
		Type:	[morph	[stem				]]
		Precedence:	1	2				
	b.	Stem:		[[s’sŭmm		]	-osi	]
		Type:		[[stem		]	morph	]
		Precedence:		1			2	
	c.	Min stem:		[s’s-	-ŭmm	]		
		Type:		[morph	morph	]		
		Precedence:		1	2

In this example, each of the stems is included in a separate record, which look like the following. The ContainedIn field is a foreign key field which must contain a value. The maximal stem in (29) is not contained in any other stem; we use *NULL* to fill the “ContainedIn” and “Precedence” fields. This stem is linked to Lemma_ID lemma-0002223.

**Table:** 

(29)	Stem_ID:	stem-00004390
	ContainedIn:	*NULL*
	Precedence:	*NULL*
	LabStem:	ŭs’sŭmmosi
	LabStemCategory:	VAI
	Word_ID:	word-JT1889-7010
	Lemma_ID:	lemma-0002223

The non-maximal stems in (30) and (31) have a non-null value for the ContainedIn field.

**Table:** 

(30)	Stem_ID:	stem-00004686
	ContainedIn:	stem-00004390
	Precedence:	2
	LabStem:	s’sŭmmosi
	LabStemCategory:	VAI
	Word_ID:	word-JT1889-7010
	Lemma_ID:	lemma-0002436

**Table:** 

(31)	Stem_ID:	stem-00004745
	ContainedIn:	stem-00004686
	Precedence:	1
	LabStem:	s’sŭmm
	LabStemCategory:	VTA
	Word_ID:	word-JT1889-7010
	Lemma_ID:	lemma-0001888

#### Morphemes

Each morpheme is assigned a unique Morpheme_ID. Because all morphemes are contained in a Stem, the morpheme record includes a Stem_ID, which is a foreign key linking to the Stem record. The Precedence field contains a number which counts the linear order of the morpheme with respect to any sister stems and morphemes within the same constituent. The LabMorpheme and LabMorphemeCategory fields both have “Lab” prefixes because they represent an analysis by the lab. The LabMorphemeCategory consists of a finite number of categories, which follow the traditional Algonquian position-based template (Bloomfield, 1946; Goddard, 1990). Some of these categories (e.g. initials, medials) correspond to *roots* in computational linguistics, because they are bound, morphologically unanalyzable forms, from which stems and words may be derived via affixation (Sproat, 1992, p. 249). Other categories (e.g. finals, preverbs, prenouns) are *bound morphemes*. This set of categories is still in flux, but we give a few typical examples below. The Lemma_ID contains a foreign key linking to a record within the Lemma table. The Morpheme record does not contain a “translation” field; since all translations of morphemes are abstract, we opted to put the translation into the Lemma record.

- Morpheme_ID: Unique key for each stem entry.
- Stem_ID: [foreign key that links to the stem the morpheme is contained in]
- Precedence: For any morpheme, this field contains a number representing the linear order of the morpheme with respect to any other sister stems and morphemes.
- LabMorpheme: The form of the morpheme in each word or stem token, given in the original orthography, as analyzed by the lab.
- LabMorphemeCategory: The morpheme category or part of speech, as analyzed by the lab. Examples: init (initial), pn (prenoun), pv (preverb), med (medial), fai (animate intransitive final), fta (transitive animate final), etc.
- Lemma_ID [foreign key that links to the abstract lemma record]: This allows us to link all of the different spellings of a stem or morpheme to a single unique lemma.
- LabMorphemeComments: Any comments from lab members.

Continuing our example from Tims (1889), there is one morpheme (a preverb) contained in the maximal stem, stem-00004390. The morpheme record is given in (32).

**Table:** 

(32)	Morpheme_ID:	morph-00000686
	Stem_ID:	stem-00004390
	Precedence:	1
	LabMorpheme:	ŭ-
	LabMorphemeCategory:	pv
	Lemma_ID:	lemma-0002245

The middle stem contains a stem and another morpheme (a final), (33). We have specified the precedence of the final as “2” (to come after the stem, which is “1”). Alternatively, we could derive the precedence order of finals by script, because finals will always be last in a stem.

**Table:** 

(33)	Morpheme_ID:	morph-00000690
	Stem_ID:	stem-00004686
	Precedence:	2
	LabMorpheme:	-osi
	LabMorphemeCategory:	fai
	Lemma_ID:	lemma-0002240

And there are two morphemes contained in the minimal stem, shown in (34) and (35). Here, we have specified the precedence order, but these could also possibly be determined by script, because initials always occur before finals.

**Table:** 

(34)	Morpheme_ID:	morph-00000691
	Stem_ID:	stem-00004745
	Precedence:	1
	LabMorpheme:	s's-
	LabMorphemeCategory:	init
	Lemma_ID:	lemma-0002313

**Table:** 

(35)	Morpheme_ID:	morph-00000692
	Stem_ID	stem-00004745
	Precedence:	2
	LabMorpheme:	-ŭmm
	LabMorphemeCategory:	fta
	Lemma_ID:	lemma-0002227

The Stems and Morphemes tables capture some of the hierarchical structure within the word. Each stem or morpheme record can also be seen as particular instances of an abstract lemma. We turn to the Lemmas table next.

#### Lemmas

The records in the Lemmas table are types which abstract over multiple tokens of the same stem or morpheme, across multiple sources and orthographies. In other words, a stem or a morpheme is an instance or example of a lemma. A stem or morpheme may contain variation due to many factors, such as orthographic choices, dialect, time period, the skill of the transcriber, etc. The lemma is always given in a standardized form (in LabLemma) based on Frantz's (1978) orthography without any diacritics, including those that indicate pitch accent or stress. As discussed at the end of Sect. 3.1, stress is largely predictable in verbs (Weber, 2016, 2020), and serves a low functional load in nouns. Although the location of stress on nouns is not predictable, stress very rarely distinguishes nominal minimal pairs (Frantz, 2017, p. 3; Stacy, 2004). The location of stress also varies within a word depending on the morphological components within that word (Kaneko, 1999; Stacy, 2004; Weber, 2016). Rather than make assumptions about which stresses are lexical and which are not, we opted to omit stress information from the lemmas entirely. Information about stress is still available in individual word tokens, whose stems are linked to the Lemmas table. We also include an abstract translation of the lemma in LabLemmaTranslation, and an abstract category in LabLemmaCategory. The LabLemmaTranslation field allows the lab to provide a correct, English translation, even if the translations in the original source were incorrect or in another language. The problem of inaccurate translation exists for other databases working with older records as well, such as the Indigenous Languages Digital Archive (ILDA; developed from the Miami-Illinois Digital Archive [MIDA] and documented in Baldwin et al., 2016). ILDA provides a corrected word translation as part of the word record, but we found it more useful to provide all lab corrections in the lemma records. This way, the original translation remains in the word record in connection to the OriginalWord, but the Lemmas table provides a standardized, corrected translation. The LabLemmaTranslation may be empty. The reason is that some finals are abstract and only carry information about valency and stem type. These will have a LabLemmaCategory, but no LabLemmaTranslation in particular.

- Lemma_ID: Unique key for each abstract lemma entry
- LabLemma: A standardized version of the stem or morpheme, written in a modern orthography with no diacritics.
- LabLemmaTranslation: A standardized, abstract version of the translation.
- LabLemmaCategory: An abstract category, if applicable.
- LabLemmaComments: Any comments from lab members.

The reason we include a category for lemmas is because sometimes the category of a lemma varies across instances of a stem or a morpheme. For example, there is a suffix *-hkaa* ‘get, acquire’, which derives either VAI or VII verb stems (Dunham, 2009; Frantz, 2017, p. 118). Consequently, each instance of the morpheme would have a LabMorphemeCategory of either “fai” or “fii”, depending on how it was used or translated in context. The LabLemmaCategory would instead be “fai/fii”. Similarly, the root *maan-* ‘recent, new, young’ can be used within a stem (LabMorphemeCategory = init) or as a preverb to a stem (LabMorphemeCategory = pv). That means that the LabLemmaCategory would be “init/pv”. This provides a way to see which constituents are always a particular category, and which may vary.

Continuing with the example from Tims (1889) above, there are seven Lemmas in total: three stems, and four morphemes. The three stem Lemmas are shown in (36)–(38).

**Table:** 

(36)	Lemma_ID:	lemma-0002223
	LabLemma:	aissammohsi
	LabLemmaTranslation:	‘s.o. sees themself’
	LabLemmaCategory:	VAI

**Table:** 

(37)	Lemma_ID:	lemma-0002436
	LabLemma:	ssammohsi
	LabLemmaTranslation:	‘s.o. see themself’
	LabLemmaCategory:	VAI

**Table:** 

(38)	Lemma_ID:	lemma-0001888
	LabLemma:	ssamm
	LabLemmaTranslation:	‘s.o. see s.o.’
	LabLemmaCategory:	VTA

And the four morpheme Lemmas are shown in (39)–(42).

**Table:** 

(39)	Lemma_ID:	lemma-0002245
	LabLemma:	a-
	LabLemmaTranslation:	imperfective
	LabMorphemeCategory:	pv

**Table:** 

(40)	Lemma_ID:	lemma-0002240
	LabLemma:	-ohsi
	LabLemmaTranslation:	reflexive
	LabMorphemeCategory:	fai

**Table:** 

(41)	Lemma_ID:	lemma-0002313
	LabLemma:	ss-
	LabLemmaTranslation:	thus
	LabLemmaCategory:	init

**Table:** 

(42)	Lemma_ID:	lemma-0002227
	LabLemma:	-amm
	LabLemmaTranslation:	watch, look at
	LabLemmaCategory:	fta

## Methods

In this section we describe our methods for data entry, with a focus on how we ensured consistency across a large team. Methods and workflow were fairly consistent for each source. Lab members identified sources from 1743 to 2017 with substantial Blackfoot words and phrases. We focused on linguistic reference materials, including dictionaries, grammars, and wordlists. However, other types of sources could be easily added in the future, including glossed narratives and time-aligned transcriptions of audio or video recordings.

We used an iterative process in which the output of each phase feeds into the work of the next phase. There are four phases:

- Phase 0: Discovery (of sources)
- Phase 1: Digitization (of each source)
- Phase 2: Analysis of maximal stems and lemmas (of each source)
- Phase 3: Analysis of maximal stems into stems, morphemes, and lemmas (across multiple sources)

Later phases of this process often affected earlier analyses. For example, we might discover that we misanalyzed a stem in one source only after comparing stems across multiple sources. Because of this, we imported data into the MySQL database in batches at particular points in the analysis: after Phase 2 and after Phase 3. This is described in more detail below.

### Phase 0: Discovery

The resources were compiled from several different places. Most sources were known from summaries of previous work, especially the annotated bibliography in Taylor (1969, pp. 8–22). This list was augmented by some sources mentioned in Hayden (1863, pp. 253–255) and the bibliography in Murdock (1941, pp. 70–72). Some sources were found by chance in the process of locating other sources; this was the case with Isham (1949/1743), which was found via Taylor (1992).

Due to the COVID-19 pandemic, many services at the Yale Library were halted or disrupted. We were able to access digital copies of the majority of sources via HathiTrust's Emergency Temporary Access Service. When availability is disrupted, this service allows authorized member library patrons to obtain digitized items in HathiTrust that correspond to physical books held by their own library. Many other sources were obtained from the Internet Archive (https://archive.org/), especially pre-1900 public domain sources, and JSTOR, especially post-1900 sources.

### Phase 1: Digitization

Lab members typically worked with digital scans of resources that did not have Optical Character Recognition (OCR) applied to them. We found this to be preferable, even though it meant that research assistants had to type each lexical item manually rather than copying and pasting from a scan. The reason is that OCR tends to render specialized characters and images of hardcopy books inaccurately; those inaccuracies are then embedded in the pdf and would be copied into the database itself.

We used Google Sheets to digitize the words and other information from each source. There were many advantages to using this platform. Google Sheets allows synchronous viewing and editing by multiple people in different locations, as well as rudimentary version control which we found necessary for such a large collaboration. The platform was already familiar to the undergraduate research assistants, which meant that no training was required to use it. We were able to begin digitization immediately, even before the database structure and software were finalized. The database structure emerged in response to the data and its inherent challenges. The flexible, easily alterable format of the spreadsheets allowed us to change and add fields in the middle of the digitization process as we adapted the database structure to the wide variety of sources we encountered.

We created several tools to help the lab members work asynchronously and independently. We created a template sheet which contained columns for each of the fields in the Words table. This was continuously updated as the database structure changed. We also created a master task list which listed the steps for digitization. Lab members updated this task to show which task they were working on for each source, and again to show that they had completed the task. This way, lab members were never duplicating work and the lab director had an overview of how far along in the process we were for each source.

To enter data, lab members copied the template for each individual source and entered information about the lexical forms. Technically, all sources could have been entered into a single Sheet, but in practice we found that very large Sheets took a long time to load. It was simpler to split the Words table by source. Each row included a lexical form (usually a phonological inflected word) and its associated data. If the original source included a phrase, we split the phrase into individual inflected words, one per row. This task was trivial for sources which used consistent punctuation marks, such as spaces or hyphens, to separate words. Some sources mark every syllable break identically, regardless of whether this occurs at a word boundary or not. In those cases we determined word boundaries based on linguistic analysis. The WordTranslation, if not provided, was determined by comparing to related forms within the database, and via linguistic analysis.

The lab had eight to twelve undergraduate research assistants at any one time. With a lab that large, even if each student entered data in an internally consistent way, there were inconsistencies across students.^17^ One example of this type involved how we handled sources who give two morphologically unrelated words with a single translation. Rather than entering two words into a single cell, we decided to put one lexical form in each row and copy the translation. This creates a database in “first normal form” (Dimitriadis & Musgrave, 2009), with one piece of information per cell. But it did require feedback and training to achieve a consistent format, and sometimes we had to go back and correct previous work once we realized that there were inconsistencies. In addition, mistakes in transcription on a project this size are inevitable. To ensure consistency, we documented our decisions about data entry, and all sources were double-checked by a different lab member than the one who initially digitized the information. This meant that each source always passed through two different hands: the first pass to digitize the source, and a second pass by a different person to double-check that all forms had been entered correctly. After this, the lab director would often do a third pass to remove inconsistencies in data entry between lab members. Once all words from a source were digitized and checked, all words were given a unique Word_ID key.

### Phase 2: Analysis of maximal stems and lemmas

We determined the maximal stems and their categories in the following way. We created a new Google Sheet which would hold all the forms in the database. Into this Sheet we copied the Word_ID, OriginalWord, and WordTranslation from each completed source, and then created other columns which correspond to the fields in the Stems table. The maximal stem was created from the OriginalWord by removing inflectional prefixes and suffixes and placing the remainder into the LabStem column. This required some amount of training for the undergraduate research assistants. Once these values were added, they were double-checked by a second lab member before a final check by the lab director. The stem class was filled into the LabStemCategory column based on evidence from the inflectional morphology, and each stem was given a unique Stem_ID. Eventually, the Stems columns were copied into a new Stems Sheet, which would become the Stems table. None of these maximal stems have any value in the ContainedIn column. On import into the MySQL database all empty ContainedIn fields would be given a *NULL* value.

We also added columns for the Lemma_ID, LabLemma, and LabLemmaTranslation. These would later be moved to a separate Sheet. The LabLemma was determined by examining paradigms of related forms in the Stems Sheet. There is a real question here about how abstract the LabLemma should be: too abstract and it is likely to be closer to an internal reconstruction, but if the LabLemma reflects some of the more recent innovations in Blackfoot then the similarities to other languages will be lost. For now our goal has been to make sure that all related allostems are “tagged” with the same LabLemma, since that is how we mark that stems are instances of the same lemma in the database. The precise form of the LabLemma can always be changed later.^18^ The LabLemmaTranslation was created from the WordTranslation by replacing each subject and object with either “s.o.” or “s.t.” (short for “someone” and “something,” two common abbreviations used respectively for animate and inanimate arguments in Algonquian linguistics). This sometimes resulted in a single LabLemma being listed together with multiple different translations, so a second pass was needed to standardize the LabLemmaTranslations. Once this was completed, each unique lemma was given a unique Lemma_ID, and the Lemmas columns were copied into a new Lemmas Sheet, which would become the Lemmas table after duplicate entries were removed.

Once several sources had reached the end of Phase 2, we exported the words, maximal stems, and lemmas associated with those sources to.csv files and imported those into the MySQL database structure. The data from Stage 2 will feed into our analysis in Stage 3.

### Phase 3: Analysis of non-maximal stems, morphemes, and lemmas

At the time of writing, we are still in the midst of Phase 2 for many sources. Once all maximal stems have been analyzed and added to a single Stems spreadsheet (a still ongoing process), the stems will be broken down into morphemes and stems along with their associated lemmas, in a manner similar to Phase 2. The use of having all maximal stems in one Sheet is that the Sheet can be sorted alphabetically by LabStem. This means that the morphemes at the left edges of the stem will be sorted together, making them much easier to find and remove. In this way, we will analyze the morphemic structure of the stems bit by bit in a recursive fashion, by removing the left edge morpheme and then re-sorting to find the next. We expect the final set of LabStemCategory and LabMorphemeCategory values to be finalized during this process. This is an advantage yet again of working in Google Sheets, where values are easily changed.

Embedded stems will be added as a new line to the stems spreadsheet, and the value for their “ContainedIn” field will be the Stem_ID of the stem they are contained in. The stem will be given a precedence number based on its linear order within the containing stem. The LabLemma and LabLemmaTranslation will be created at the same time as the stems in the Stems Sheet in a similar manner as Phase 2. The lemmas will later be standardized, given a Lemma_ID, and copied into the Lemmas Sheet along with the others.

Morphemes will be added to a separate Morphemes Sheet, with a foreign key to the stem they are contained in. The morphemes will be given a precedence number based on their linear order within the containing stem. The morpheme lemmas will be created in the Morphemes Sheet at the same time in a similar manner as before. The lemmas will later be standardized, given a Lemma_ID, and copied into the Lemmas Sheet along with the others.

Once all stems, morphemes, and lemmas have been analyzed, they will be exported in batch to.csv files. The existing Stems, Morphemes, and Lemmas tables in the MySQL database will be cleared, and the full.csv files will be re-imported. It is important that no words, stems, or lemmas change their unique key during this process, or else the links between records will be lost.

### Overview of sources and phases

Table 5 below gives an overview of the sources in the database and where they are in this iterative process.

**Table 5:** Current phase for each sources

Reference	Phase 1	Phase 2	Phase 3
Isham (1949/1743)	✓	✓ (v1)	✓ (v1.1)
Umfreville (1790)	✓	✓ (v1)	✓ (v1.1)
Franklin (1823)	✓	✓ (v1)	✓ (v1.1)
Hale (1846)	✓		
Catlin (1842)	✓		
Latham (1846)	✓	✓ (v1)	✓ (v1.1)
Gallatin (1848)	✓	✓ (v1)	✓ (v1.1)
Hale (1848)	✓		
Howse (1849)	✓		
Schoolcraft (1854)	✓		
Hayden (1863)	✓		
Morgan (1871)	✓		
Lanning (1882)	✓		
Hale (1886)	✓	✓ (v1)	✓ (v1.1)
Lacombe (1886)	✓		
Wilson (1888)	✓		
Tims (1889)	✓		
Maclean (1896)	✓		
Curtis (1911)	✓	✓ (v1)	✓ (v1.1)
Geers (1917)	✓		
Schultz (1926)	✓		
Uhlenbeck and van Gulik (1930)	✓		
Uhlenbeck (1938)	✓		
Voegelin (1941)	✓		
Schultz (1962)	✓		
Taylor (1967)	✓	✓ (v1)	✓ (v1.1)
Taylor (1969)	✓	✓ (v1)	✓ (partial; v1.1)
Frantz (1971)	✓		
Holterman (1996)	✓		
Frantz (2017)	✓		
Frantz and Russell (2017)	✗		

## Future linguistics projects

The database was created with several potential projects in mind, ranging from linguistic analysis to community language projects of various kinds. Below, we focus on some of the linguistic research projects for which this database is particularly well-suited. We also believe the database could support projects in language maintenance and pedagogy, but because the authors of this paper are linguists, these fall outside our particular expertise. We plan to involve the Blackfoot communities in decisions about future project directions.

### Dialects and variation

The sources in the database represent a diverse set of dialects and time periods, which presents the opportunity to analyze stability and change within Blackfoot. The four Blackfoot dialects are mutually intelligible, but contain lexical, morphological, and phonological differences that are salient to speakers (Bliss & Ritter, 2009; Bliss & Gick, 2009, 2017; Bliss & Glougie, 2010; Frantz, 2017, ch. 1; Peter, 2014: 14*ff*; Wissler, 1911: 8). However, “there is as much variation between speakers from the same reserve as there is between speakers from different reserves” (Frantz & Russell, 2017, p. *xiii*), and generational differences in pronunciation and grammar also cross-cut the dialects (Bortolin & McLennan, 1995; Kaneko, 1999; Miyashita & Chatsis, 2013; Van Der Mark, 2003). As Genee and Junker (2018) point out, Blackfoot variation within and across dialects is under-documented and not studied in a systematic fashion, although the new “Documenting variation in Niitsi’powahsin (Blackfoot)” project (PIs: Inge Genee & Marie-Odile Junker; funded by a SSHRC Insight Grant) aims to fill this gap.

We have recorded dialect and variation in several ways across the database. Dialect information is included in each source or word record, when known. Each lemma links to all instances or examples of the lemma (e.g. each stem or morpheme), which provides a way to check for variation of that lemma. This requires the researcher to first view all instances of the lemma together before determining whether there is variation in the lemma at all. A useful addition in the future might be to include tags in the Lemmas table for common types of variation, so that these can be studied more easily.

There are some types of variation which are difficult to capture in our database, such as differences in lexical semantics or category across dialects, as documented in Frantz and Russell (2017). For example, there are cases where one word has different meanings in different dialects. For instance, the stem *áípakkohtamm* means ‘tractor’ in the Káínai (Blood) dialect, but ‘motorcycle’ in the Aapátohsipikani (Northern Peigan) dialect.^19^ There are also cases where a noun belongs to the animate noun class in one dialect, but to the inanimate noun class in another. For instance, the stem *iitáísapahtsimao’p* ‘ashtray’ is animate in the Káínai (Blood) dialect, but inanimate in the Aapátohsipikani (Northern Peigan) dialect. Because each distinct combination of phonological/orthographic form, translation, and category is treated as a distinct Lemma, identical phonological forms with differences in meaning or category within and across dialects will be listed under different Lemmas. Of course, a targeted search over the LabLemma field (e.g. for ‘*aipakkohtamm*’ or ‘*iitaisapahtsimao’p*’) would clearly show the variation in meaning or noun class across dialects. A more robust solution might be to add a table of standardized glosses. (See Bowern, 2016 for another implementation of standardized glosses.) In these examples, the variation would be apparent when searching by keyword, since the keyword ‘tractor’ would link to *áípakkohtamm* (Káínaa) and *áípakkohsoyi* (Peigan), and the keyword ‘ashtray’ would link to the animate stem in the Káínaa dialect and the inanimate stem in the Peigan dialect.

### Historical change

Because this database makes the lexical forms from many different sources available, it presents a unique opportunity to study Blackfoot historical phonology. Blackfoot has often been called “divergent” with respect to Algonquian (most recently in Goddard, 2018), in part because of the substantial number of phonological innovations and substantial neutralization of contrasts (Berman, 2006, 2007; Proulx, 1989, 2005; Thomson, 1978; Weber, 2017). These changes can make cognates difficult to find, and a complete reconstruction of sound changes in Blackfoot remains elusive. The timespan of the sources in the database is deep enough that it might contain written evidence of some recent sound changes in the language, though these may be obscured by the fact that our sources used dramatically different notation methods. It is also possible that the earlier sources record a language variety (a “doculect”, to use a term from Cysouw & Good, 2013) which maintains some contrasts which have since neutralized.

The database contains lexical forms from such a great time depth, that it could also help clarify the relationship of Blackfoot to the other Algonquian languages, which is also debated. Blackfoot is one of the so-called “Plains Algonquian” languages, an areal grouping which includes Cheyenne, Arapaho and Gros Ventre (Atsina), but any shared features within this group have been argued to be the result of contact rather than shared innovations (Goddard, 1994; Mithun, 1999). Early researchers doubted that Blackfoot was an Algonquian language at all (Franklin, 1823; Gallatin, 1836; Howse, 1849; Mackenzie, 1789). Gallatin (1848) was the first to lay out a significant number of cognates to other Algonquian languages, although his work was not based on systematic sound correspondences. Later research confirmed that Blackfoot is related to other Algonquian languages based on putative word and morpheme cognates (Berman, 2006, 2007; Goddard, 2018; Michelson, 1935; Proulx, 1989, 2005; Taylor, 1960; Thomson, 1978; Uhlenbeck, 1924; Weber, 2017) as well as shared inflectional systems (Bliss et al., 2010; Goddard, 2018; Michelson, 1912; Morgan, 1966; Voegelin, 1941) and shared derivational morphemes (Déchaine & Weber, 2015, 2018). Goddard has argued more recently based on certain archaic retentions that Blackfoot is the oldest dialectal layer of Algonquian (Goddard, 1994), or even a sister to the Algonquian family (Goddard, 2018).

Diachronic change is not directly captured in the database, but each lemma links to all instances or examples of the lemma (e.g. each stem or morpheme), which provides a way to check for historical change or archaisms. This requires the researcher to first view all instances of the lemma together before determining whether there is diachronic change in the lemma at all. A useful addition in the future might be to include an InternalReconstruction field in the Lemmas table. This should make the task of building correspondence sets across Algonquian and finding cognates with Blackfoot much easier.

### Derivational and inflectional morphology

The database focuses on lexical forms with various amounts of complexity, including words, stems, and morphemes. This means that the database could be used to study phonologically-conditioned allomorphy of forms, as well as other aspects of morphosyntax. Unfortunately, there is no current consensus in how to analyze complex words into constituent parts. Recent reference materials (Frantz, 2017; Frantz & Russell, 2017), do not always discuss the internal structure of complex stems, and there exists no list of Blackfoot initials, medials, and finals. Older reference materials such as Geers (1917), Uhlenbeck (1938), and Taylor (1969) discuss some internal structure, but their analyses do not always align. More recent linguistic research has also discussed the internal structure of stems, but again, the analyses do not always converge (Armoskaite, 2011; Genee et al., 2012; Weber, 2020, 2022, *forthcoming*). There are also multiple phonological processes which occur at morpheme boundaries (segment deletion and epenthesis, vowel coalescence, etc.) which have been factored into previous analyses to a greater or lesser extent. For example, Weber (2021, *forthcoming*) points out that some epenthetic vowels at morpheme boundaries have been incorrectly analyzed as part of a morpheme in some sources. This is another way that existing analyses are not reliable or accurate.

As described in Sect. 5, one aspect of the Blackfoot Words database involves analysis of lexical forms via tokenization, phonemicization, and lemmatization. This has already resulted in new morphemic analyses of Blackfoot stems and lemmas, which we expect to contribute to related projects, such as the Blackfoot Digital Dictionary (https://dictionary.blackfoot.atlas-ling.ca/), part of the Niitsi'powahsin (Blackfoot Language) Resources website; Nisinoon (https://nisinoon.net/), a cross-linguistic database of Algonquian stem-internal morphemes; and the Database of Algonquian Language Structures (https://alglang.net/). In the future it may be possible to use the data in Blackfoot Words to model derivational morphology computationally. This has already been done for Plains Cree (Arppe et al., 2019).

Currently, inflectional affixes are not encoded in Blackfoot Words at all, unless they were documented in the PartialWord fields from an analysis by one of the original source authors. In fact, each stem is determined by *removing* inflectional affixes. Incorporating this information into the database would create a rich dataset which could be of use to academic researchers as well as language learners.

There are several ways this information could be integrated in the future. One option is to add an Inflections table, where each inflection record would include a foreign key to the word or stem they are contained in. Each inflection would also contain a foreign key to a lemma in the Lemmas table. Another option would be to include fields in the Words and Stems tables which would allow us to tag each record for specific inflectional information (e.g. the person, number, and animacy features of the subject and object, clause type, etc.) Either of these options would create a way for researchers to search for all inflected words with either a specific inflectional affix or specific set of grammatical properties.

This information could also be used to train morphological parsers, which have many potential applications. For example, a parser could take a complex inflected word as an input and automatically analyze and translate it, which could be useful for the present project as well as for language learners. A parser could also take a stem as an input and generate inflectional paradigms, which could then be displayed in an online dictionary, such as the Blackfoot Digital Dictionary. Dunham (2014) created two morphological parsers which use Finite State Transducers (FST) to model the complex morphophonology of Blackfoot; it is possible that these could be modified for our use. There are other examples of FST parsers for Algonquian languages: for Cree see Arppe et al. (2017) and Harrigan et al. (2017); for Arapaho see Kazeminejad et al. (2017). The current Plains Cree FST parser is available on github (https://giellalt.github.io/lang-crk/), and is already implemented in itwêwina (https://itwewina.altlab.app/) and the Plains Cree online dictionary search engine (https://dictionary.plainscree.atlas-ling.ca/#/help).

### Expansions

We have tried to create a flexible database structure so that the project can expand in different directions. Although we have focused on published documentation, the database could easily be expanded to include new types of sources, such as lexical items from audio or video annotations or stories from various publications and repositories. Especially important would be literature written by Blackfoot speakers, which would be a good supplement to the lexicographic and grammatical works written by non-native speakers documenting the language. In many cases these stories have been interlinearized and glossed, which can already be accommodated in the PartialWords fields.

It would be trivial to add additional fields to the existing tables. For example, we could add fields to the Words, Stems, and Morphemes tables which contains an automatically-generated standardized form based on the mappings between the orthographic keys we recorded. This standardized orthography would not include sounds that were not represented in the original source orthography. However, it would create uniformity from the many different systems of vowel transcription, which would make it easier to compare stems and morphemes across sources.

It would also be possible to add additional tables to support the research projects above. A Variation table could contain types of phonological, morphological, and semantic variation. This would link to the Words table in a many-to-many relationship (e.g. a word record could link to many records in the Variation table, and a variation record could link to many records in the Words table). A Cognates table would be one way to include putative cognates across the Algonquian family; each record in the table would contain a foreign key to a record in the Lemmas table. And as we mentioned above, inflectional affixes could easily be entered into a new Inflections table and linked to words and stems in the database.

However, a neater method of expansion might use Application Programming Interface (API) queries to dynamically include relevant data from other existing databases, such as the Blackfoot Digital Dictionary, Nisinoon, the Database of Algonquian Language Structures, or others. In this way, the Blackfoot Words project would be enriched by other projects even while it contributes to them. In short, we developed the database structure with these research projects in mind so that we have the option of adding more functionality in the future.

### Community involvement

Our plan is to involve the Blackfoot communities in the project before creating future versions of the database. There are several reasons for this. First, there is growing recognition that ethical research involving Indigenous languages means co-designing speech and language technologies with communities (Bird, 2020; Liu et al, 2022; Walter & Suina, 2019). However, the initial version of Blackfoot Words was created by linguists and students without the involvement of the Blackfoot communities. Second, following the Indigenous Data Sovereignty movement, we believe that the Blackfoot communities have the right to determine the means of collection, access, analysis, interpretation, management, dissemination and reuse of Blackfoot language data (Kukutai & Taylor, 2016; Snipp, 2016; Walter & Suina, 2019). Although we did not collect the language data in the database ourselves, we currently determine how that data is managed, disseminated, and reused. Third, Blackfoot Words was created with several research goals in mind, as outlined in this section, but could potentially support community-based projects as well as other resources like the Blackfoot Digital Dictionary. Creating those synergies requires community involvement and support.

Several questions regarding how to expand the database have already arisen which require community input. For example, some resources include taboo words or translations which are now considered derogatory. We do not wish to make decisions on whether to include these without first consulting the Blackfoot communities. There are also many possible ways to present the data depending on user preference, which may affect the underlying database structure or our choices in how to move to a more permanent API. There are already models of collaborative projects between linguists and Blackfoot community members (Bliss et al, 2021; Fish & Miyashita, 2017; Genee & Junker, 2018; Kipp et al., 2015; Miyashita & Chatsis, 2013; Miyashita & Crowshoe, 2009; Mizumoto & Genee, 2018), which can serve as a model for our collaboration in the future.

## Conclusion

The paper describes the initial publication of Blackfoot Words, a database of lexical forms in Blackfoot. The lexical forms are taken from language documentation (grammars, dictionaries, and wordlists) which span nearly 300 years and all four major dialects. Working with this type of corpus presents some unique challenges. For example, the corpus contains a plethora of orthographic notations, some of which are not internally consistent or do not capture all the phonemic sounds of the language. The analyses and translations by the original source authors are often incorrect or missing key information. And finally, the lexical forms often contain other stems and morphemes, but the internal structure of stems in Blackfoot is largely unknown. It is difficult or impossible to determine lemmas of the internal components of these lexical forms without first determining their internal structure.

The database structure is designed to address some of these challenges, and we also use the data in the corpus to help us determine a morphemic analysis of each of the forms. We keep the original source material separate from any analyses imposed on the data by the lab. That is, the records for each digitized lexical form and the records for corrected, standardized lemmas and other analyses are held in separate tables. In addition, the database structure captures the hierarchical structure of stems and morphemes within each word record, while also linking those elements to abstract, standardized lemmas. We spend some time discussing our methods, especially how we ensured consistency by requiring separate processes of data entry and data checking by different people. We end by discussing several research projects that the database is uniquely suited to support, now or in the future.

## Data Availability

The database can be freely viewed at https://www.blackfootwords.com/. In addition, most of the underlying source materials are in the public domain.

## Footnotes

1 The database can be viewed at https://www.blackfootwords.com/. The underlying MySQL database is published under a Creative Commons license and can be downloaded at https://doi.org/10.5281/zenodo.5774980.

2 https://dictionary.blackfoot.atlas-ling.ca/.

3 https://www.onlinelinguisticdatabase.org/.

4 The stem is given in square brackets, []. Abbreviations which are not included in the Leipzig Glossing Rules (Comrie, Haspelmath, & Bickel, 2015) are as follows: ai=animate intransitive, ii=inanimate intransitive, ind=independent order, liquid=liquid material, ta=transitive animate, ti=transitive inanimate. We adopt the practice here of using hyphens on affixes and stem-internal elements to indicate “boundedness”. Stems are written without hyphens, despite the fact that they must be inflected to form a complete word.

5 Some secondary finals are discussed in Frantz (2017: 111*ff*) under the name “concrete finals”.

6 The Algonquian family extends across North America from British Columbia to Labrador and as far south as North Carolina (Mithun, 1999). It is well-established by lexical reconstructions based on regular sound changes (Bloomfield, 1925, 1946; Michelson, 1935; Siebert, 1941, 1975; Aubin, 1975; Pentland, 1979; Hewson, 1993).

7 The spelling is usually ‘Peigan’ in Canada, but ‘Piegan’ in the United States. We use ‘Piegan’ when referring to both Piegan Nations together.

8 Note that the English dialect name ‘Blood’ is not a direct translation of the Blackfoot ‘Káínai’. Káínai comes from Aakáínaa, which means Many Chiefs (Bastien, 2004: 9–10).

9 https://blackfootconfederacy.ca/.

10 In this paper we refer to the language as Blackfoot.

11 Umfreville's vocabularies were inaccurately copied in Graham (1969), as discussed in Pentland (1975). Because of this, we have included the Blackfoot wordlist from Umfreville (1790) but not from Graham (1969) in the database.

12 The Anglican missionary John William Tims (1889) created a Blackfoot syllabary, which is still used by some families today (e.g. Ermineskin and Howe, 2005; Áístainskiaaki et al., 2013), but this syllabary was not used by any of the sources included thus far.

13 Frantz (2017: 1*ff*) refers to centralized and peripheral vowels as “lax” and “tense”, respectively. We avoid that terminology here because of the associations between laxness and unstressed syllables in some languages.

14 For a helpful introduction to linguistic databases written for linguists, see Dimitriadis and Musgrave (2009).

15 Note that our definition of ‘stem’ for the database is a superset of the grammatical stem described in Sect. 3.2. We discuss this in more detail in Sect. 5.2.3.

16 We opted for a term like “partial words” rather than a linguistic term like “constituents” because these entries are not always a well-formed morpho-syntactic constituent.

17 An anonymous reviewer points out that the quality of annotation will vary between annotators and that this can be valuable information both when training computational models on the data and for linguistic inquiry. One way to mitigate this would be to include in each table unique annotator IDs, which have been shown elsewhere to impact the performance of machine learning models (Geva et al., 2019). One downside of using Google Drive is that the version control features are limited and this type of information is not automatically captured. We plan to include annotator ID's in future versions of the database.

18 It is likely that we need two fields to reflect both goals: a LabLemma field which reflects a modern, standardized version of the stem, and an InternalReconstruction field, which reflects an internal reconstruction. This type of addition can be easily accommodated in the database structure.

19 The stem for ‘tractor’ is *áípakkohsoyi* in the Aapátohsipikani (Northern Peigan) dialect, and the word for ‘motorcycle’ is *áípakkitaka’siwa* in the Káínai (Blood) dialect.

## Code availability

The MySQL database can be downloaded from https://doi.org/10.5281/zenodo.5774980.
